# Reoviruses hijack the SMARCB1-MYC transcriptional regulation complex to activate autophagy for persistent viral infection in leafhopper vector

**DOI:** 10.1371/journal.ppat.1013569

**Published:** 2025-10-09

**Authors:** Hui Wang, Runfa Liu, Guangming Xiao, Yanan Li, Bozhong Li, Qian Chen, Taiyun Wei

**Affiliations:** State Key Laboratory of Agricultural and Forestry Biosecurity, Fujian Agriculture and Forestry University, Fuzhou, Fujian, China; University of Kentucky, UNITED STATES OF AMERICA

## Abstract

Autophagy plays a crucial role in virus-host interactions, as viral components and particles can be degraded by the host’s autophagic machinery. Additionally, some viruses can hijack autophagy for their own benefit. However, the mechanisms underlying the transcriptional regulation of autophagy by arboviruses in insect vectors remain largely unexplored. In this study, we found that rice dwarf virus (RDV) infection activates the autophagy pathway in the leafhopper vector, *Nephotettix cincticeps*, and this autophagy activation also facilitates viral infection in the leafhopper. We identified that MYC transcription factor regulates the expression of autophagy proteins ATG5 and ATG8 by directly targeting their promoters. A transcription regulator SMARCB1 binds to MYC and impedes its recognition of the ATG5 and ATG8 promoters, thus negatively regulating their expression. Moreover, NcSMARCB1 negatively regulates ATG5 expression by directly binding to its promoter. RDV major outer capsid protein P8 blocks the nuclear translocation of SMARCB1, disrupting the SMARCB1-MYC interaction and thereby relieving the transcriptional inhibition of ATG5 and ATG8, which leads to autophagy activation. Furthermore, major outer capsid protein P8 of rice gall dwarf virus (RGDV), same to RDV belonging to plant reoviruses, also interacts with SMARCB1 in leafhopper *Recilia dorsalis*, preventing its nuclear translocation. Similarly, suppression of SMARCB1 expression enhances autophagy formation and promotes RGDV infection. These findings highlight the critical role of insect vector SMARCB1 and MYC in regulating autophagy in response to arbovirus infection.

## Author summary

Autophagy plays a dual role in arbovirus-insect vector interactions, either degrading viral components or being exploited by viruses. Here, we reveal that rice dwarf virus (RDV) activates autophagy in its leafhopper vector *N. cincticeps*, promoting viral infection. The MYC transcription factor upregulates ATG5 and ATG8 expression, while SMARCB1 suppresses autophagy by blocking MYC’s binding to these promoters and directly inhibiting ATG5 transcription. RDV’s capsid protein P8 disrupts SMARCB1 nuclear translocation, relieving MYC repression and activating autophagy. Similarly, rice gall dwarf virus (RGDV) hijacks SMARCB1 in *R. dorsalis*, enhancing autophagy and infection. These findings demonstrate a conserved mechanism by which plant reoviruses manipulate autophagy through SMARCB1-MYC regulation in insect vectors.

## Introduction

Autophagy is a conserved cellular self-protection process that helps cells maintain homeostasis by degrading cellular materials or invading pathogens [1]. It is tightly regulated at multiple levels, including transcriptional, post-transcriptional, and post-translational [2,3]. Autophagy initiates with the formation of double-membraned autophagosomes, which subsequently fuse with lysosomes to form autolysosomes, leading to the degradation of engulfed cellular components [4–6]. This process is tightly regulated by autophagy-related (ATG) genes. ATG4 processes nascent ATG8 by cleaving its C-terminal arginine, generating ATG8-I. ATG8-I then conjugates with phosphatidylethanolamine (PE) to form ATG8-II, a critical mediator of autophagosome membrane expansion and a well-established autophagosomal marker [5,7]. The ATG12-ATG5 conjugate acts as an E3-like enzyme which is required for lipidation of ATG8 family proteins and their association to the vesicle membranes [4,5]. The autophagic receptor SQSTM1/p62, which interacts with ATG8, is selectively degraded during autophagy, serving as a reliable indicator of autophagic flux [5]. In contrast, lysosomal-associated membrane protein 1 (LAMP1) accumulates upon autophagosome-lysosome fusion, reflecting lysosomal engagement in the degradative process [8].

The expression of many autophagy-related (ATG) genes is maintained at a low level under healthy conditions and is substantially up-regulated in response to nutrient starvation and pathogens infection [9,10]. This rapid induction of ATG gene expression is essential for optimal autophagy [11]. Autophagy biogenesis can be transcriptionally regulated by chromatin remodeling complex and transcription factors such as microphthalmia/transcription factor E (MiT/TFE) family, Forkhead box-containing protein O, Relish and SMARCB1 [12–14]. MYC, a member of the basic helix-loop-helix leucine zipper (bHLH-Zip) transcription factor family, is involved in multiple cellular processes, including DNA repair, protein translation, cell cycle arrest, stress response, cellular proliferation and differentiation, programmed cell death, immune response regulation and autophagy [15,16]. MYC is referred to as a “master gene regulator” and regulates approximately 15% of the human genome [15]. MiT/TFE family includes microphthalmia-associated transcription factor (MITF), transcription factor E3 (TFE3), transcription factor EB (TFEB) and transcription factor EC (TFEC) proteins, also belonging to the bHLH-Zip family, that can recognize a specific motif (GTCACGTGAC) known as the coordinated lysosomal expression and regulation [17,18]. SMARCB1 is a core component of the SWI/SNF (BAF) chromatin remodeling complex and plays a prominent role in cell proliferation and differentiation, in cellular antiviral activities and inhibition of tumor formation [13]. It has also been reported that SMARCB1, as a transcription factor, directly binds to ATG5 promoter in myeloma to inhibit ATG5 expression and thus regulate autophagy [13]. Although many transcription factors involved in autophagy have been identified, the mechanisms by which pathogens regulate autophagy at the transcriptional level remain largely unknown.

During virus infection in insect vectors, autophagy can act either as an antiviral process by degrading viral proteins and particles or as a facilitator of viral propagation and spread when ‘manipulated’ by the virus [19]. For example, tomato yellow leaf curl virus (TYLCV) or rice black-streaked dwarf virus (RBSDV) activates antiviral autophagy in their insect vectors [20,21]. In contrast, the rice gall dwarf virus (RGDV) and southern rice black-streaked dwarf virus (SRBSDV) induces the pro-viral autophagy and exploits autophagosomes for viral propagation and dissemination in insect vector [22–24]. Plant viruses activate autophagy in their insect vectors through various mechanisms. Rice black-streaked dwarf virus (RBSDV) virions or outer capsid protein P10 promote phosphorylation of AMP-activated protein kinase (AMPK) in the vector *Laodelphax striatellus*, leading to the phosphorylation of glyceraldehyde 3-phosphate dehydrogenase (GAPDH) [21]. The phosphorylated GAPDH then translocated from the cytoplasm to the nucleus, activating the autophagy pathway in the insect midgut cells [21]. In the vector *Sogatella furcifera*, SRBSDV bind the viral receptor integrin β3 on the plasma membrane of the midgut epithelial cells, stimulating the c-Jun N-terminal kinase (JNK) signaling pathway. This leads to upregulate expression of the ATG3, ATG5, ATG8, and ATG12, which are directly involved in inducing autophagy in SRBSDV-infected *S. furcifera* [24]. In the vector *Recilia dorsalis*, rice gall dwarf virus (RGDV) P2 expression alone can induce autophagy through interaction among P2, GAPDH and ATG4B [25]. In addition, viral nonstructural protein Pns11 directly recruits ATG5-ATG12 conjugation by binding ATG5 on the phagophore membranes to induce the formation of autophagosomes [26]. Thus, RGDV proteins interact directly with vector proteins to promote autophagosomes formation [26]. These findings demonstrate that the capsid protein or nonstructural protein of arboviruses play a key role in activating autophagy in insect vector cells by different ways, but how viruses affect the transcriptional regulation of ATG genes in insect vector remains unclear.

Rice dwarf virus (RDV, *Phytoreovirus*, *Reoviridae*) is transmitted by the green rice leafhopper *Nephotettix cincticeps* in a persistent-propagative manner and is transovarially transmitted [27]. The RDV genome encodes seven structural proteins (P1, P2, P3. P5, P7. P8 and P9) and five nonstructural proteins (Pns4, Pns6, Pns10, Pns11 and Pns12). Among these proteins, P8 is the major outer capsid protein, while Pns6, Pns11 and Pns12 are the components of the viroplasm [27,28]. RDV enters leafhopper cells through receptor-mediated, clathrin-dependent endocytosis using P2 as the viral attachment molecule [29]. Furthermore, RDV is proposed to exploit tubular structures composed of the nonstructural protein Pns10 (Pns10 tubules) to move along actin-based filopodia extending toward neighboring cells, thereby enhancing intercellular viral propagation among cultured leafhopper cells [27]. More importantly, two obligate symbiotic bacteria, *Sulcia* and *Nasuia*, in *N. cincticeps* harbor RDV particles through interaction of viral capsid protein and bacterial outer membrane proteins, facilitating transovarial viral transmission and thus forming a virus-bacterium synergistic interaction [30,31]. Previous studies have reported that the persistent replication of RDV in cultured *N. cincticeps* cells can trigger the autophagy process, as evidenced by the presence of obvious virus-containing double-membraned autophagosomes and the conversion of ATG8-I to ATG8-II [22]. In this study, we confirm the autophagy pathway triggered by RDV infection facilitates viral propagation in the leafhopper vector *N. cincticeps*. We further revealed that the NcSMARCB1 has a dual function in decreasing the transcription level of ATG5 by binding the ATG5 promoter and inhibiting the NcMYC’ ability to bind to promoters of ATG5 and ATG8, thus disrupting the autophagy activation and mediating the antiviral response against RDV infection. However, RDV P8 binds to NcSMARCB1 and blocks its nuclear translocation, resulting in the inhibition of NcSMARCB1-mediated transcription suppression of autophagy. Similarly, RGDV P8 can also interact with RdSMARCB1 and interfere with its nuclear translocation, thus activating autophagy for it’s persistent transmission. This suggests that SMARCB1 plays a crucial role in insect vector defense against reoviruses which utilize viral proteins to target this key component of autophagy and promote infection.

## Results

### 1 Autophagy pathway induced by RDV infection facilitated viral infection in *N. cincticeps*

**Fig 1:**
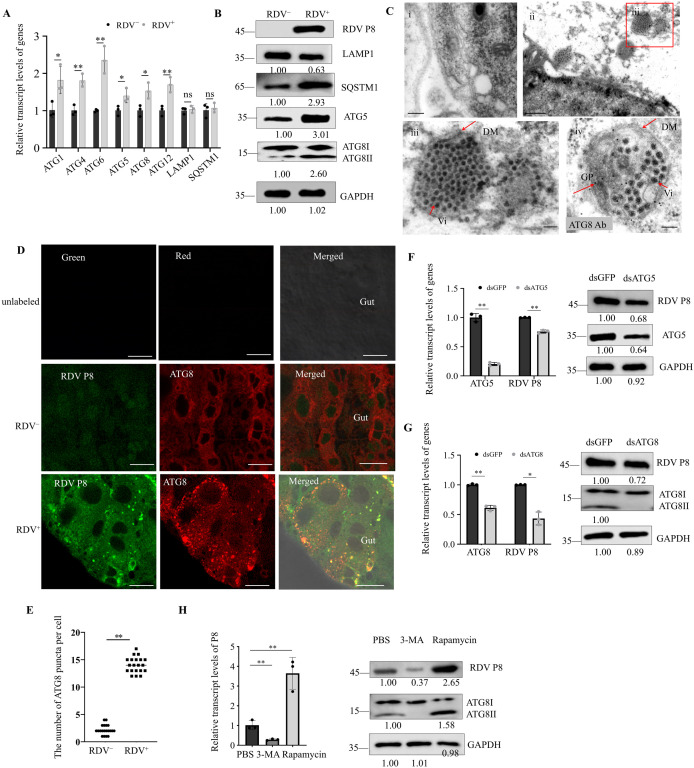
RDV infection activated the autophagy pathway in *N. cincticeps.* (A) RT-qPCR showing the relative transcript of ATG1, ATG4, ATG5, ATG6, ATG8, ATG12, LAMP1 and SQSTM1 genes in nonviruliferous and RDV-infected *N. cincticeps*. (B) Western blot assays showing the protein levels of ATG5, ATG8, LAMP1 and SQSTM1 genes in nonviruliferous and RDV-infected *N. cincticeps*.Insect GAPDH served as a control. (C) Transmission electron micrographs showing RDV-induced autophagosomes in the midgut epithelial cells. (i) The midgut epithelium of nonviruliferous *N. cincticep*. (ii) RDV-containing autophagosomes within virus -infected midgut epithelium. Panel B (iii) is an enlargement of the boxed area in ii. (iv) Immunogold labeling of ATG8 in RDV-containing autophagosomes. RDV-infected intestines of *N. cincticeps* were immunolabeled with ATG8 specific IgG as the primary antibody, followed by treatment with 15-nm gold particle-conjugated IgG as the secondary antibody. Vi: virion; DM: double membrane; GP: gold particle. Bars, 200 nm (i); 500 nm (ii); 100 nm (iii, iv). (D) Immunofluorescence microscopy showing the localization of RDV P8 and autophagosomes in nonviruliferous and RDV-infected *N. cincticeps* midgut epithelial cells. The dissected intestines from nonviruliferous and RDV-infected *N. cincticeps* were immunolabeled with RDV P8 and ATG8 antibodies. Unlabeled intestines are shown as a control. Bars, 500 μm. (E) The number of ATG8 puncta in the midgut epithelial cells of nonviruliferous and viruliferous leafhoppers intestinal tissues are shown in D. **P < 0.01. (F, G) RT-qPCR and western blot assays showing the relative transcript and protein levels of ATG5, ATG8 and RDV P8 in dsGFP, dsATG5 (F) or dsATG8 (G)-treated RDV-infected *N. cincticeps.* Insect GAPDH served as a control. (H) RT-qPCR and western blot assays showing the relative transcript and protein levels of ATG and RDV P8 in RDV-infected *N. cincticeps* treated with (+) and without (-) 3-MA or rapamycin. Insect GAPDH served as a control. Data in A, F-H are presented as means (± SD) of three independent biological replicates and each replicate contains 30 insects (two-tailed t test). *, *p* < 0.05; **, *p* < 0.01; ns, not significant. The proteins in B, F-H were detected by western blot assay using indicated antibodies. The relative intensities of the bands for these proteins are shown below using Image J. Shown is a western blot of one out of three biological replicates and each replicate contains 30 insects.

We extracted total RNA from nonviruliferous and RDV-infected *N. cincticeps* to determine the relative mRNA levels of ATG genes using RT-qPCR. The results showed that the relative mRNA levels of ATG1, ATG4, ATG5, ATG6, ATG8 and ATG12 increased significantly in viruliferous *N. cincticeps*, while SQSTM1 and LAMP1 remained unchanged (Fig 1A). Total proteins from viruliferous and nonviruliferous *N. cincticeps* were extracted and western blot assay showed that ATG8-II increased in viruliferous *N. cincticeps* compared to nonviruliferous controls, suggesting that RDV infection facilitates the conversion of ATG8-I to ATG8-II (Fig 1B)*.* Furthermore, the accumulation levels of ATG5 and SQSTM1 were increased, while LAMP1 was decreased in viruliferous *N. cincticeps*. To further verify the activation of the autophagy pathway in RDV-infected *N. cincticeps*, we observed the formation of autophagosomes in the gut using transmission electron microscopy and immunofluorescence microscopy. Transmission electron microscopy showed the presence of virus-containing autophagosomes in RDV-infected midgut epithelium of *N. cincticeps*, but not in nonviruliferous control (Fig 1C). Immunoelectron microscopy showed that ATG8 antibody also specifically labeled these autophagosomes (Fig 1C). Thus, RDV particles can be engulfed by virus-induced autophagosomes in *N. cincticeps*. Immunofluorescence microscopy showed that minimal ATG8 labeling in the intestines of nonviruliferous *N. cincticeps* (Fig 1D). At four days post-first access to diseased plants (padp), we observed that ATG8-specific autophagosomes colocalized with RDV P8 in the intestine epithelium of viruliferous *N. cincticeps* (Fig 1D). There was a significant increase in the average number of ATG8 puncta per infected cell compared with those in the uninfected cells (Fig 1E). Taken together, our results demonstrated that RDV infection induced the incomplete autophagy within insect vectors.

To confirm whether the autophagy pathway influences viral infection in the *N. cincticeps*, we suppressed the expression of ATG5 and ATG8 by RNAi and then measured the level of P8 gene of RDV in *N. cincticeps*. RT-qPCR and western blot assay showed that the RDV P8 in dsATG5- or dsATG8-treated viruliferous leafhoppers decreased than those in dsGFP-treated controls at 4 dapd (Fig 1F, G). The transmission efficiency on rice plants of dsATG5- and dsATG8-inhibited leafhoppers was 26.00% and 22.00%, respectively, compared with 44.33% after dsGFP injection (S1A Fig). Our results suggested that the inhibiting of autophagy by dsATG5 or dsATG8 treatment decreased RDV accumulation in *N. cincticeps*. To further observe whether autophagy promotes RDV infection in *N. cincticeps*, we checked whether RDV P8 accumulation and protein expression could be modulated by activating autophagy with rapamycin or inhibiting autophagy with 3-MA. After rapamycin or 3-MA treatment, *N. cincticeps* were allowed to feed on RDV-infected rice plant for 24 h and then transferred to healthy rice plant. ATG8-II can be specifically detected in rapamycin-treated *N. cincticeps*, suggesting that autophagy was induced after rapamycin treatment for 24 h. The RDV P8 in rapamycin-treated *N. cincticeps* was significantly increased than that of untreated populations, indicating that viral infection was promoted by autophagy (Fig 1H). The transmission efficiency by leafhoppers treated with rapamycin was 60.67%, compared with 46.67% after PBS injection (S1B Fig). In contrast, 3-MA-treated *N. cincticeps* blocked the formation of ATG8-II in *N. cincticeps* and inhibited the accumulation of RDV P8 (Fig 1H). The transmission efficiency by insects treated with 3-MA was 20.67% and, compared with 46.67% after PBS injection (S1B Fig). Taken together, these results indicate that the RDV-induced autophagy pathway facilitates viral infection and transmission by *N. cincticeps.*

### 2 NcMYC regulates ATG5 and ATG8 expression by directly targeting the promoter of ATG5 and ATG8

**Fig 2:**
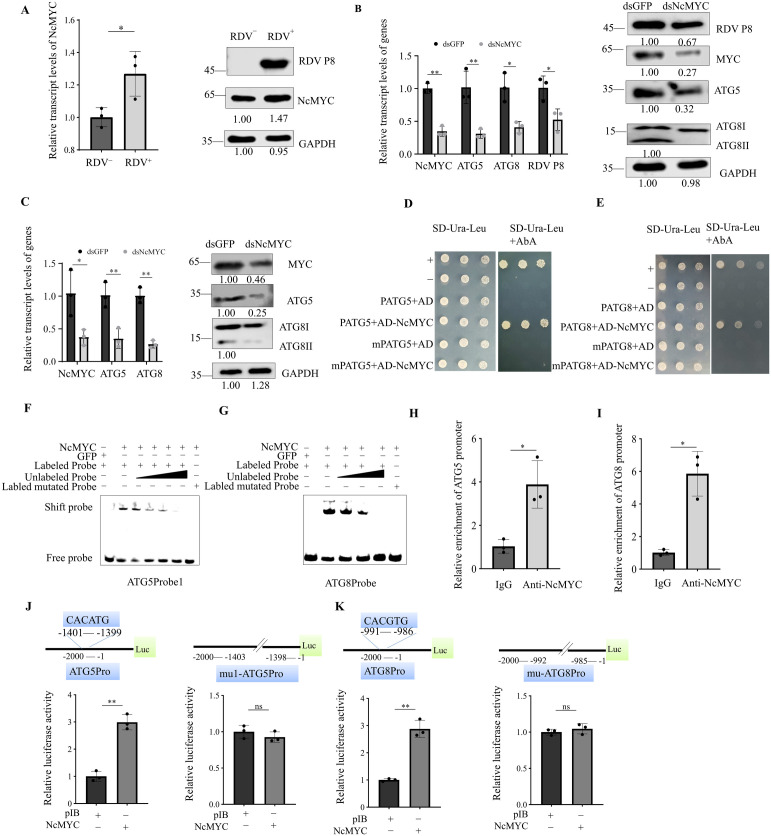
NcMYC regulates ATG5 and ATG8 expression by directly targeting the promoter of ATG5 and ATG8. (A) RT-qPCR and western blot assays showing the relative transcript and protein levels of NcMYC in nonviruliferous and RDV-infected *N. cincticeps*. (B) RT-qPCR and western blot assays showing the relative transcript and protein levels of NcMYC, ATG5, ATG8 and RDV P8 in dsGFP or dsNcMYC-treated RDV-infected *N. cincticeps*. (C) RT-qPCR and western blot assay showing the relative transcript and protein levels of ATG5 and ATG8 in dsGFP or dsNcMYC-treated nonviruliferous *N. cincticeps*. Data in A-C are presented as means (± SD) of three independent biological replicates and each replicate contains 30 insects (two-tailed t test). *, *p* < 0.05; **, *p* < 0.01; ns, not significant. The proteins were detected by western blot assay using indicated antibodies. Insect GAPDH served as a control in A-C. The relative intensities of the bands for these proteins are shown below using Image J. Shown is a western blot of one out of three biological replicates and each replicate contains 30 insects. (D, E) Y1H assay showing that NcMYC binds to the promoter sequence of ATG5 (D) and ATG8 (E). The full-length coding sequence of NcMYC was cloned into the vector pGADT7 (AD-NcMYC). The promoters of ATG5 and ATG8 were cloned into the vector pABAi (PATG5 and PATG8). The mutated ATG5 and ATG8 promoter sequences (CAC[A/G]TG mutated to CAAAAA) were cloned into the vector pABAi (mPATG5 and mPATG8). The transformed yeast cells were incubated on SD/-Trp-Ura and SD/-Trp-Ura + AbA medium. (F, G) EMSA showing the binding ability of purified NcMYC to Cy5-labeled probes of ATG5 (F) and ATG8 (G) promoter. Shift probes showing the binding of NcMYC to the Cy5-labeled probes of the ATG5 (“CACATG”-containing sequences) and ATG8 (“CACGTG”-containing sequences) promoter. The unlabeled probe with the increased concentrations was added as a competitor. The mutated ATG5 and ATG8 promoter probe is a probe with “CAAAAA”-containing sequences (CAC[A/G]TG mutated to CAAAAA). (H, I) ChIP-qPCR assay showing that NcMYC binds to the promoter sequence of ATG5 and ATG8. Chromatin from *N. cincticeps* were immunoprecipitated by anti-MYC antibody and amplified with ATG5 and ATG8 promoter-specific primers. IgG was used instead of the anti-MYC antibody in the immunoprecipitation step as the control. Data are presented as means (± SD) of three independent biological replicates and each replicate contains 100 insects (two-tailed t test). *, *p* < 0.05; **, *p* < 0.01; ns, not significant. (J, K) The dual luciferase reporter assays showing that NcMYC binds to the promoter of ATG5 and ATG8. The pGL3-ATG5, pGL3-mu1-ATG5 (CACATG mutated to CAAAAA) (J) or pGL3-ATG8, pGL3-mu-ATG8 (CACGTG mutated to CAAAAA) (K) promoter was transfected with pIB-NcMYC into Sf9 cells. pIB empty vector was used as a control. The cells were harvested at 60 h after transfection, and the luciferase activities were measured by normalizing to the REN signals.

In order to unravel the transcriptional regulators regulating ATGs, ATG8 promoter was used to construct a yeast one-hybrid (Y1H) bait for screening a cDNA library of *N. cincticeps*. Among the sequenced clones, one candidate was annotated as transcription factor MYC. NcMYC contained 1614 nt and encoded 537 amino acid residues, which had a canonical bHLHzip_Myc domain at its C-terminus (S2A Fig). RT-qPCR and western blot assays showed that the NcMYC expression level was significantly up-regulated in the RDV-infected *N. cincticeps* (Fig 2A). We then suppressed NcMYC expression by microinjecting *N. cincticeps* with synthesized dsNcMYC or dsGFP to investigate how dsNcMYC influence the infection of RDV. After the insects were microinjected with dsMYC or dsGFP, the dsMYC‐treated leafhoppers and control leafhoppers did not apparently differ physiologically, and both had survival rates over 70% at 8 d post dsRNAs treatment (S3A Fig). RT-qPCR and western blot assays showed that the knockdown of NcMYC expression effectively decreased ATG5, ATG8 and RDV P8 accumulation in *N. cincticeps* (Fig 2B). The transmission efficiency by insects injected with dsNcMYC was 23.33% (average of 28, 22, and 20%), compared with 44% (average of 50, 36, and 46%) after dsGFP injection (S1C Fig). Thus, RDV can increase expression of NcMYC in *N. cincticeps* to benefit the accumulation and transmission of RDV.

We next investigated whether NcMYC regulates ATG genes in nonviruliferous *N. cincticeps*. RT-qPCR and western blot assays confirmed that ATG5 and ATG8 were downregulated by microinjection of dsNcMYC in nonviruliferous *N. cincticeps* (Fig 2C). Sequence analysis revealed that ATG5 and ATG8 promoter contains a “CACATG” and “CACGTG” motif, respectively, which are canonical *cis*-acting elements recognized by MYC [32]. To confirm the interaction between NcMYC and ATG5/ATG8 promoter, we first performed a point-to-point Y1H assay, using NcMYC as a prey and the bait vector constructed suing the ATG5/ATG8 promoter. Yeast cells co-transformed with the prey and the bait vectors grew normally on the selective media, similar to that of the positive control. When “CAC[A/G]TG” motif was mutated, the yeast cells failed to survive on the selection medium containing Aureobasidin A (AbA) (Fig 2D, E), implying that NcMYC can bind to “CAC[A/G]TG” motif in the promoter. We then employed electrophoretic mobility shift assay (EMSA) to test the binding of NcMYC to the “CAC[A/G]TG” *cis*-acting elements in vitro. NcMYC directly bound to the ATG5 and ATG8 promoter sequences containing “CAC[A/G]TG” (Fig 2F, G). However, NcMYC did not bind to the mutated sequences of “CAC[A/G]TG” (CAC[A/G]TG mutated to CAAAAA) by EMSA (Fig 2F, G). To further verify the interaction between NcMYC and promoter of ATG5 and ATG8 in vivo, chromatin immunoprecipitation (ChIP) assay with anti-MYC antibody was carried out using leafhopper. The following qPCR analysis showed that NcMYC was enriched in the ATG5 and ATG8 promoter region in *N. cincticeps* (Fig 2H, I). To investigate whether the transcription factor NcMYC directly activates the transcription of ATG5 and ATG8, we performed a dual luciferase assay. Sf9 cells were co-transfected with the NcMYC expression vector and the luciferase reporter constructs containing the 2-kb promoter region of ATG5 and ATG8 (ATG5pro-Luc and ATG8pro-Luc). The activity of the ATG5pro-Luc and ATG8pro-Luc reporter increased significantly compared to cells transfected with empty expression vectors (Fig 2J, K). Next, site-directed mutation in the “CAC[A/G]TG” motif was made to construct the mu1-ATG5pro-Luc (CACATG mutated to CAAAAA) and mu-ATG8pro-Luc reporter plasmid (CACGTG mutated to CAAAAA). Co-transfection of mutated reporter plasmid with NcMYC did not increase transcription activity significantly (Fig 2J, K). Collectively, these results demonstrate that NcMYC regulates ATG5 and ATG8 expression by directly targeting the promoter of ATG5 and ATG8.

### 3 NcSMARCB1 negatively regulates ATGs expression by directly targeting the promoter of ATG5 and binding with NcMYC

**Fig 3:**
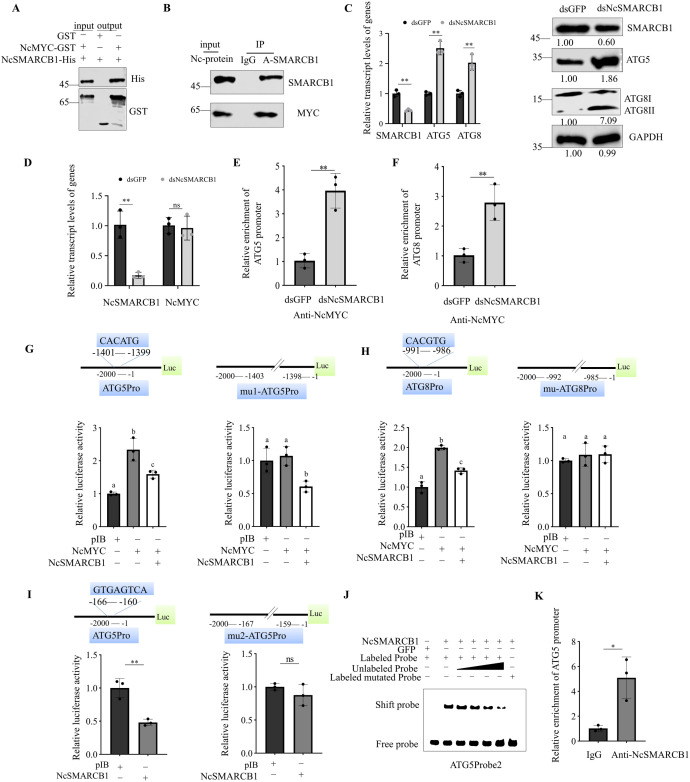
NcSMARCB1 inhibits RDV accumulation in leafhopper by negatively regulates ATG5 expression. (A) GST pull-down assay showing the interaction of NcSMARCB1 with NcMYC. (B) Co-IP assay showing the interaction of NcSMARCB1 with NcMYC. (C) RT-qPCR assay and western blot assays showing the relative transcript and protein levels of ATG5 and ATG8 in dsGFP or dsNcSMARCB1-treated nonviruliferous *N. cincticeps*. (D) RT-qPCR assay showing the relative transcript levels of NcMYC in dsGFP or dsNcSMARCB1-treated nonviruliferous *N. cincticeps*. (E, F) ChIP-qPCR assay showing that relative enrichment of ATG5 (E) and ATG8 (F) promoter binding with NcMYC in dsGFP or dsNcSMARCB1-treated nonviruliferous *N. cincticeps*. The comparable amounts of NcMYC precipitated from dsGFP or dsNcSMARCB1-treated nonviruliferous *N. cincticeps* and relative enrichment of ATG5 promoter were quantified qPCR assay. (G, H) NcSMARCB1 inhibits the transcriptional activity of NcMYC. The pGL3-ATG5, pGL3-mu1-ATG5 (CACATG mutated to CAAAAA) or pGL3-ATG8, pGL3-mu-ATG8 (CACGTG mutated to CAAAAA) promoter was transfected with NcMYC alone or together with NcSMARCB1 into Sf9 cells. The cells were harvested at 60 h after transfection, and the luciferase activities were measured by normalizing to the REN signals. (I) The dual luciferase reporter assays showing that NcSMARCB1 binds to the promoter sequence of ATG5. The pGL3-ATG5, or pGL3-mu2-ATG5 (GTGAGTCA mutated to GTTTTTAA) promoter was transfected with pIB-NcSMARCB1 into Sf9 cells. pIB empty vector was used as a control. The cells were harvested at 60 h after transfection, and the luciferase activities were measured by normalizing to the REN signals. (J) EMSA showing the binding ability of purified NcSMARCB1 to Cy5-labeled probe2 of ATG5 promoter. Shift probes showing the binding of NcSMARCB1 to the Cy5-labeled probe of the ATG5 promoter (“GTGAGTCA”-containing sequences). The unlabeled probe with the increased concentrations was added as a competitor. The mutated probe is a probe with “GTTTTTAA”-containing sequences (GTGAGTCA mutated to GTTTTTAA). (K) ChIP-qPCR assay showing that NcSMARCB1 binds to the promoter sequence of ATG5. Chromatin from *N. cincticeps* were immunoprecipitated by anti-SMARCB1 antibody and amplified with ATG5 promoter-specific primers. IgG was used instead of the anti-SMARCB1 antibody in the immunoprecipitation step as the negative control. Data in C-F, K are presented as means (± SD) of three independent biological replicates and each replicate contains 30 in RT-qPCR assay, 100 in ChIP-qPCR assay (two-tailed t test). *, *p* < 0.05; **, *p* < 0.01; ns, not significant. The proteins in C were detected by western blot assay using indicated antibodies. Insect GAPDH served as a control. The relative intensities of the bands for these proteins are shown below using Image J. Shown is a western blot of one out of three biological replicates and each replicate contains 30 insects.

SMARCB1 binds directly to the carboxy-terminus of MYC and alters MYC function in human cell [33]. The full-length ORF of NcSMARCB1 contained 1,113 nt and encoded 370 amino acid residues including a WH_NTD_SMARCB1 domain and SNF5 domain (S2B Fig). Similarly, we found that NcSMARCB1 interacts with NcMYC by glutathione S-transferase (GST) pull-down and Co-IP assays (Fig 3A, B). Compared with the dsGFP-treated group, the transcript levels of ATG5 and ATG8 were significantly higher in dsNcSMARCB1-treated *N. cincticeps* (Fig 3C), suggesting that NcSMARCB1 is involved in the negative regulation of ATG5 and ATG8 expression. Furthermore, we examined the transcript level of NcMYC after suppressing the expression of NcSMARCB1 in *N. cincticeps*. There was no change in NcMYC transcript levels in dsSMARCB1-treated *N. cincticeps* compared to the dsGFP-treated group (Fig 3D). To investigate whether NcSMARCB1 interferes with the transcription function of NcMYC, we performed ChIP-qPCR to compare the binding of NcMYC to the ATG5 and ATG8 promoters in dsGFP-treated and dsNcSMARCB1-treated *N. cincticeps*. The results showed that significantly more ATG5 and ATG8 promoter DNA was bound by NcMYC in dsNcSMARCB1-treated *N. cincticeps* compared to the dsGFP-treated group (Fig 3E, F). These results indicated that NcSMARCB1 interfered with the binding of NcMYC to the ATG5 or ATG8 promoter in leafhopper.

To confirm whether NcSMARCB1 interferes with the binding of NcMYC to the ATG5 or ATG8 promoter, we performed a dual-luciferase reporter assay to dissect the role of NcMYC and NcSMARCB1 in regulating ATG5 and ATG8 expression. The reporter activities of ATG5pro-Luc and ATG8pro-Luc were markedly inhibited by the co-expression of NcSMARCB1 and NcMYC compared to cells transfected with NcMYC alone (Fig 3G, H). However, the reporter activities of mu-ATG8pro-Luc (CACGTG mutated to CAAAAA) did not change with the co-expression of NcSMARCB1 and NcMYC compared to cells transfected with NcMYC alone (Fig 3H). EMSA assay further confirmed that NcSMARCB1 interferes the binding of NcMYC and promoters of ATG5 (S4A Fig) and ATG8 (S4B Fig).

Interestingly, the reporter activities of mu1-ATG5pro-Luc (CACATG mutated to CAAAAA) were markedly inhibited by the co-expression of NcMYC and NcSMARCB1, compared to cells transfected with NcMYC alone (Fig 3G), suggesting that NcSMARCB1 may directly bind to the ATG5 promoter. The dual luciferase assay was performed to investigate whether the NcSMARCB1 directly inhibits the transcription of ATG5. The activity of the ATG5pro-Luc decreased compared to cells transfected with empty expression vectors (Fig 3I). Next, site-directed mutation was made to construct the mu2-ATG5pro-Luc (GTGAGTCA mutated to GTTTTTAA) reporter plasmid and the co-transfection of mutated reporter plasmid with NcSMARCB1 did not decrease transcription activity significantly (Fig 3I). On the contrary, EMSA showed that NcSMARCB1 directly bound to the ATG5 promoter sequences containing “GTGAGTCA” (Fig 3J). However, NcSMARCB1 did not bind to the mutated sequences of “GTTTTTAA” (GTGAGTCA mutated to GTTTTTAA) (Fig 3J) and ATG5 promoter sequences containing “CACATG” (S5 Fig). ChIP-qPCR assays showed that NcSMARCB1 was enriched in the NcATG5 promoter region in *N. cincticeps* (Fig 3K). All results showed that NcSMARCB1 also negatively regulates ATG5 expression by directly targeting the promoter of ATG5.

### 4 RDV P8 attenuates the function of NcSMARCB1 by blocking its nuclear translocation

**Fig 4:**
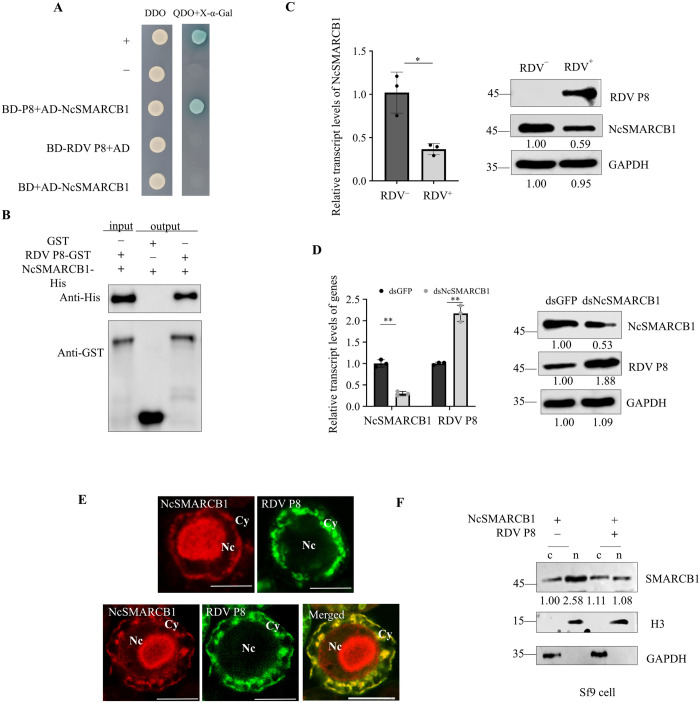
NcSMARCB1 interacts with RDV P8. (A) Y2H assay showing the interaction of NcSMARCB1 with RDV P8. Positive control, pGBKT7-53/pGADT7-T; Negative control, pGBKT7-Lam/pGADT7-T. Yeast colonies exhibit blue color upon protein interaction. (B) GST pull-down assay showing the interaction of NcSMARCB1 with RDV P8. (C) RT-qPCR and western blot assays showing the relative transcript and protein levels of NcSMARCB1 in nonviruliferous and RDV-infected *N. cincticeps.* (D) RT-qPCR and western blot assays showing the relative transcript levels of NcSMARCB1 and RDV P8 in dsGFP or dsNcSMARCB1-treated RDV-infected *N. cincticeps*. (E) Immunofluorescence assay showing the distribution of NcSMARCB1 and RDV P8 in Sf9 cells. Sf9 cells singly expressing NcSMARCB1-HA, RDV P8-His, co-expressing NcSMARCB1-HA and RDV P8-His were immunolabeled with His-FITC (green), or HA-rhodamine (red). Bars, 10 μm. (F) Nuclear-cytoplasmic separation assay showing the nuclear (n) and cytoplasmic (c) distribution of NcSMARCB1 in Sf9 cells singly expressing NcSMARCB1, co-expressing NcSMARCB1 with RDV P8. The H3 and GAPDH were used as reference proteins for nuclear and cytoplasmic proteins. Data in C, and D are presented as means (± SD) of three independent biological replicates and each replicate contains 30 insects (two-tailed t test). *, *p* < 0.05; **, *p* < 0.01; ns, not significant. The proteins in C, D, F were detected by western blot assay using indicated antibodies. Insect GAPDH served as a control in C and D. The relative intensities of the bands for these proteins are shown below using Image J. Shown is a western blot of one out of three biological replicates and each replicate contains 30 insects.

During our yeast two hybrid screening of partners that may interact with RDV P8, we identified 32 putative interactors included NcSMARCB1 (S6 Fig). Further Y2H and glutathione S-transferase (GST) pull-down assays confirmed that RDV P8 interactes with NcSMARCB1 (Fig 4A, B). RT-qPCR and western blot assays showed that RDV infection significantly inhibited NcSMARCB1 expression in *N. cincticeps* (Fig 4C)*.* We then suppressed NcSMARCB1 expression by microinjecting *N. cincticeps* with synthesized dsNcSMARCB1 or dsGFP to investigate how dsNcSMARCB1 influence the infection of RDV. RT-qPCR and western blot assays showed that the knockdown of NcSMARCB1 expression effectively increased RDV P8 accumulation in *N. cincticeps* (Fig 4D). The transmission efficiency by insects injected with ds NcSMARCB1 was 63.33% (average of 58, 68, and 64%), compared with 45.33% (average of 40, 52, and 44%) after dsGFP injection (S1D Fig). Thus, RDV decreases expression of NcSMARCB1 in *N. cincticeps* to benefit its own infection.

The nuclear translocation of NcSMARCB1 is critical for regulation of its target genes. Cellular distribution analysis of singly expressed RDV P8 and NcSMARCB1 in Sf9 cells showed that nearly all RDV P8 were localized in the cytoplasm while most NcSMARCB1 localized in the nuclear. However, RDV P8 and NcSMARCB1 were co-localized in the cytoplasm in Sf9 cells co-expressing RDV P8 and NcSMARCB1 (Fig 4E). We thus tested whether the interaction between RDV P8 and NcSMARCB1 affects the nuclear translocation of NcSMARCB1. Western blot assays revealed that the amount of NcSMARCB1 in the nucleus was lower in cells co-expressing RDV P8 and NcSMARCB1 than in cells expressing NcSMARCB1 alone (Fig 4F). These results suggested that the RDV P8 blocks NcSMARCB1’s nuclear translocation.

**Fig 5:**
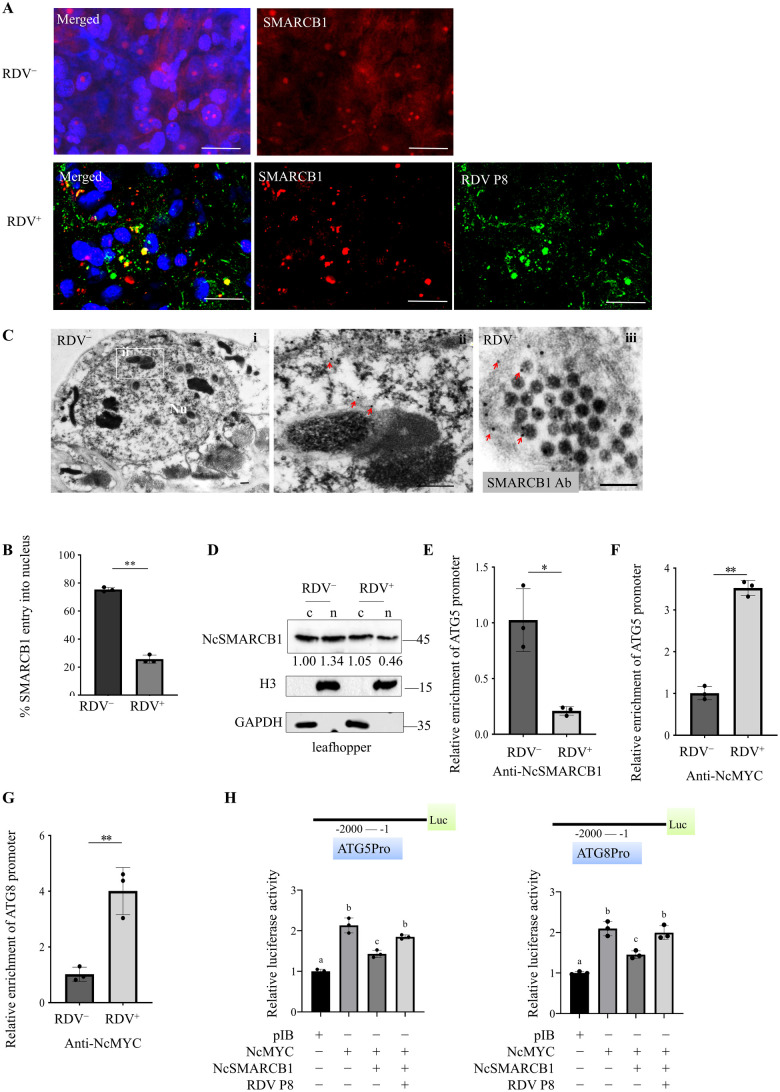
RDV P8 attenuates the function of NcSMARCB1 by blocking its nuclear translocation in *N. cincticeps.* (A) Immunofluorescence assay showing the distribution of NcSMARCB1 and RDV P8 in nonviruliferous and RDV-infected *N. cincticeps*. Nonviruliferous and RDV-infected *N. cincticeps* midgut epithelial cells were immunolabeled with RDV P8-FITC (green), NcSMARCB-rhodamine (red) and DAPI (blue). Bars, 10 μm. (B) The mean percentages of *N. cincticeps* midgut epithelial cells with NcSMARCB1 in the nucleus. Data are presented as means (± SD) of three independent biological replicates and each replicate contains 30 insects and each insect’s intestine consists of 20 cells. (two-tailed t test). **, p < 0.01. (C) Immunoelectron microscopy showing the distribution of RDV and NcSMARCB1 in nonviruliferous (i) and RDV-infected (iii) *N. cincticeps* intestines. The intestines of nonviruliferous and RDV-infected *N. cincticeps* were immunostained with SMARCB1 antibody as the primary antibody, followed by treatment with 15-nm gold particle-conjugated IgG as the secondary antibody. Panel (ii) is an enlargement of the boxed area in i. Red arrows indicate gold particles. Bars, 200 nm. (D) Nuclear-cytoplasmic separation assay showing the nuclear (n) and cytoplasmic (c) distribution of NcSMARCB1 in nonviruliferous and RDV-infected *N. cincticeps*. The H3 and GAPDH were used as reference proteins for nuclear and cytoplasmic proteins. (E) ChIP-qPCR assay showing that relative enrichment of ATG5 promoter binding with NcSMARCB1 in nonviruliferous and RDV-infected *N. cincticeps*. The comparable amounts of NcSMARCB1 precipitated from viruliferous and nonviruliferous insects and relative enrichment of ATG5 promoter were quantified qPCR assay. (F, G) ChIP-qPCR assay showing that relative enrichment of ATG5 and ATG8 promoter binding with NcMYC in nonviruliferous and RDV-infected *N. cincticeps*. The comparable amounts of NcMYC precipitated from viruliferous and nonviruliferous insects and relative enrichment of ATG5 and ATG8 promoter were quantified qPCR assay. (H) RDV P8 inhibits the transcriptional suppression activity of NcSMARCB1. The pGL3-ATG5 promoter or pGL3-ATG8 promoter was transfected with (i) NcMYC alone or (ii) NcMYC and NcSMARCB1 together or (iii) NcMYC, NcSMARCB1 and RDV P8 into Sf9 cells. The cells were harvested at 60 h after transfection, and the luciferase activities were measured by normalizing to the REN signals. Data in E-G are presented as means (± SD) of three independent biological replicates and each replicate contains 100 insects (two-tailed t test). *, *p* < 0.05; **, *p* < 0.01; ns, not significant.

We then investigated the distribution of NcSMARCB1 and RDV P8 in *N. cincticeps*. In nonviruliferious *N. cincticeps*, immunofluorescence microscopy showed the appearance of abundant NcSMARCB1 puncta in the nucleus of midgut and some diffuse NcSMARCB1 label in the cytoplasm (Fig 5A). In RDV-infected *N. cincticeps* midgut epithelial cells, immunofluorescence assay showed that RDV P8 colocalized with NcSMARCB1 puncta partially in the cytoplasm (Fig 5A). There was a significant decrease in the average number of NcSMARCB1 puncta in nucleus compared with those in the uninfected cells (Fig 5B). Immunoelectron microscopy showed that NcSMARCB1 localized to nucleus of midgut in nonviruliferious *N. cincticeps* (Fig 5C). Immunoelectron microscopy further showed that NcSMARCB1 specifically reacted with RDV virions (Fig 5C). Western blot assay revealed the amount of NcSMARCB1 in the nucleus was lower in RDV-infected *N. cincticeps* than in nonviruliferious *N. cincticeps* (Fig 5D)*,* further confirming that RDV P8 blocks NcSMARCB1 nuclear translocation in vivo. In contrast, NcMYC consistently distributed in the nucleus when singly expressed, co-expressing NcMYC and NcSMARCB1 or triply expressing NcMYC, NcSMARCB1 and RDV P8 in Sf9 cells (S7 Fig).

ChIP-qPCR assay showed that the amount of ATG5 promoter bound by NcSMARCB1 was lower in the RDV-infected *N. cincticeps* compared to nonviruliferous control (Fig 5E). Conversely, the amount of ATG5 and ATG8 promoter bound with NcMYC was higher in RDV-infected *N. cincticeps* than in nonviruliferous control (Fig 5F, G). Next, we performed a dual-luciferase reporter assay to dissect the role of NcMYC, NcSMARCB1 and RDV P8 in regulating ATG5 and ATG8 expression. Transient expression of the NcMYC effector with the reporters constructed using ATG5 or ATG8 activated the LUC activities, whereas the activation was mitigated by co-expressing NcSMARCB1 with NcMYC. However, triple-expressing NcSMARCB1, NcMYC and RDV P8 resumed the activation activity of NcMYC completely (Fig 5H). Thus, NcSMARCB1 could suppress NcMYC-activated ATGs expression, and RDV P8 liberate the NcMYC from the NcSMARCB1-NcMYC complex and recover its function in transcriptional activation.

### 5 Plant reovirus RGDV also attenuates the function of SMARCB1

**Fig 6:**
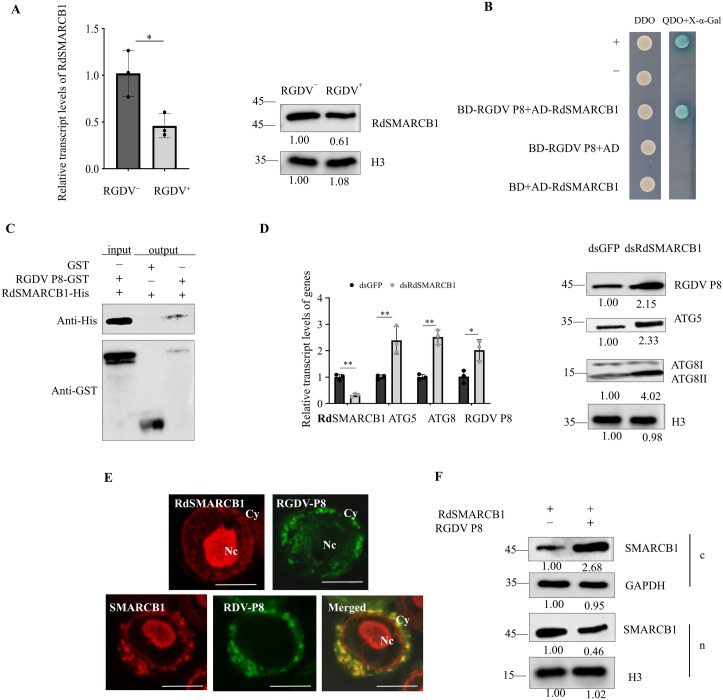
RdSMARCB1 also interacts with RGDV P8. (A) RT-qPCR and western blot assays showing the relative transcript and protein levels of RdSMARCB1 in nonviruliferous and RGDV-infected *R. dorsalis.* (B) Y2H assay showing the interactions of RdSMARCB1 with RGDV P8. Yeast colonies exhibit blue color upon protein interaction. (C) GST pull-down assay showing the interactions of RdSMARCB1 with RGDV P8. (D) RT-qPCR and western blot assays showing the relative transcript and protein levels of RdSMARCB1, RGDV P8, ATG5, and ATG8 in dsGFP or dsRdSMARCB1-treated RGDV-infected *R. dorsalis*. (E) Immunofluorescence assay showing the distribution of RdSMARCB1 and RGDV P8 in Sf9 cells. Sf9 cells singly expressing RdSMARCB1-HA, RGDV P8-His, co-expressing RdSMARCB1-HA and RGDV P8-His were immunolabeled with His-FITC (green), or HA-rhodamine (red). Bars, 10 μm. (F) Nuclear-cytoplasmic separation assay showing the nuclear (n) and cytoplasmic (c) distribution of RdSMARCB1 in Sf9 cells singly expressing RdSMARCB1, co-expressing RdSMARCB1 with RGDV P8. The H3 and GAPDH were used as reference proteins for nuclear and cytoplasmic proteins. Data in A, and D are presented as means (± SD) of three independent biological replicates and each replicate contains 30 insects (two-tailed t test). *, *p* < 0.05; **, *p* < 0.01; ns, not significant. The proteins in A, D, F were detected by western blot assay using indicated antibodies. Insect H3 was detected with H3-specific IgG as a control in A and D. The relative intensities of the bands for these proteins are shown below using Image J. Shown is a western blot of one out of three biological replicates and each replicate contains 30 insects.

To further investigate the potential role of the SMARCB1 in regulating the transcriptional of ATGs in other arbovirus and insect vector, *R. dorsalis* and RGDV were also evaluated in this study. The full-length ORF of RdSMARCB1 contained 1,125 nt and encoded 374 amino acid residues including a WH_NTD_SMARCB1 domain and SNF5 domain (S2C Fig). The results indicated that the relative expression of RdSMARCB1 was significantly downregulated when *R. dorsalis* was infected by RGDV (Fig 6A). Additionally, the RGDV P8 was found to interact with the RdSMARCB1 by the Y2H and pull-down assays (Fig 6B, C). Similar to the results of RDV, treatment of *R. dorsalis* with dsRdSMARCB1 led to a significant increase in the relative transcription levels of RdATG5, RdATG8, and RGDV P8 (Fig 6D), suggesting that RdSMARCB1 might also decrease the accumulation of ATGs and RGDV in *R. dorsalis.* Cellular distribution analysis of singly expressed RGDV P8 and RdSMARCB1 in Sf9 cells showed that nearly all RGDV P8 were localized in the cytoplasm while most RdSMARCB1 localized in the nucleus, while RGDV P8 and RdSMARCB1 were co-localized in the cytoplasm in Sf9 cells co-expressing RGDV P8 and RdSMARCB1 (Fig 6E). Western blot assay revealed that the amount of RdSMARCB1 in the nucleus was significantly lower in cells co-expressing RGDV P8 and RdSMARCB1 compared to cells expressing RdSMARCB1 alone (Fig 6F). These results suggest that RGDV P8 may activate autophagy in *R. dorsalis* by interacting with the RdSMARCB1.

## Discussion

Viruses can induce or activate cellular responses such as autophagy to facilitate their infection cycle in hosts or vectors [34]. In this study, we demonstrated that plant reovirus RDV infection triggered a significant increase in transcriptional level of ATG5 and ATG8, the colocalization of ATG8 with RDV P8, and the conversion of ATG8-I to ATG8-II in virus-infected *N. cincticeps*, indicating that autophagy pathway was activated by RDV infection in insect vector. ATG8 is localized on both the inner and outer membranes of autophagosomes and functions as an adaptor protein for selective substrates such as p62/SQSTM1 [35]. In general, autophagosome-associated ATG8 and SQSTM1 are efficiently degraded following autophagosome-lysosome fusion [36]. However, in our study, while the mRNA level of SQSTM1 remained unchanged, its protein level significantly increased, indicating impaired autophagic flux during RDV infection. A similar phenomenon was previously reported during RGDV infection in *R. dorsalis*, where SQSTM1 protein levels were elevated despite stable mRNA expression [36]. By inhibiting or activating autophagy with chemical reagents 3-MA and rapamycin, or through RNAi targeting ATG5 or ATG8 genes, we demonstrated that virus-induced autophagy pathway increased RDV accumulation in *N. cincticeps*. Thus, RDV infection activated the autophagy pathway to facilitate viral accumulation rather than controlling viral infection in the insect vector. Previous studies have showed that the plant reovirus RGDV and SRBSDV also induce the autophagy pathway, subsequently mediated nonlytic viral propagation and release from their insect vector [22–24]. Such autophagic flux is inhibited during RGDV and SRBSDV infection via blocking the fusion of virus-containing autophagosomes with lysosomes [24–26]. Previous studies also indicate that viruses like rotavirus (NSP4) and HPIV3 (phosphoprotein) disrupt autophagosome-lysosome fusion to enhance replication [37,38]. Generally, plant reoviruses in insect vector cells are sequestered in spherical vesicular compartments [39]. We deduced that the exploitation of virus-induced autophagy pathway for viral accumulation in insect vectors may be a conserved mechanism for plant reoviruses. Further studies are required to validate this hypothesis and elucidate the precise role of RDV in modulating lysosomal function.

The upregulation of ATG genes at transcriptional level after viral infection in insect vectors is common [20,21], yet the underlying mechanism is unknown. The MYC transcription factor is implicated in metabolic reprogramming, cell death, and angiogenesis in cancers [40–42]. MYC also plays a key role in autophagy [43,44]. For instance, knockdown of MYC led to decreased LC3-II/LC3-I and Atg7, as well as defects in autolysosomal degradation in osteosarcoma cells [43]. Another study reported that MYC was involved in autophagosome formation during the early stages of autophagy via the JNK1-Bcl2 pathway and ROS in HeLa cells [45]. Additionally, MYC and MYCN activated the PERK/eIF2α/ATF4 arm of the unfolded protein response (UPR) in the P493-6 human lymphoblastoid cell line and mouse embryonic fibroblast (MEF) cells, resulting in increased cell survival through the induction of autophagy [44]. In our study, RNAi experiments revealed that MYC activities the transcriptional level of ATG5 and ATG8. ChIP-qPCR and EMSA analysis further confirmed ATG5 and ATG8 are transcriptional targets of NcMYC. We uncovered the novel association between NcMYC and autophagy, suggesting another molecular mechanism by which NcMYC mediates transcription activation of ATG5 and ATG8. Previous studies demonstrated that MYC regulates the expression of multiple genes involved in diverse cellular processes [15]. Uncovering MYC’s new targets via ChIP assays may reveal previously unknown pathways underlying viral pathogenesis.

SMARCB1, also named as SNF5, BAF47, INI1, contains a conserved SNF5 domain, which can regulate the cell cycle and the development of cancers [46]. SMARCB1 does not contain nuclear localization signals (http://nls-mapper.iab.kei o.ac.jp/cgi-bin/NLS_Mapper_form.cgi), while it is a core component of the SWI/SNF (BAF) chromatin remodeling complex [13]. SMARCB1 may enter the nucleus by “hitchhiking” with histones, chromatin remodeling complexes (such as SWI/SNF), or other nuclear proteins containing nuclear localization signal (NLS). SMARCB1 modulates various oncogenic/tumour suppressor pathways by regulating of transcription through the SWI/SNF complex or through interactions with transcription factors [47]. Our study found that NcSMARCB1 interacts with NcMYC, and inhibits the binding between NcMYC and ATG5/ATG8 promoter in *N. cincticeps*. Previous studies reported that SNF5 directly binds to the carboxy-terminus of MYC, where it was originally thought to act as a co-activator of MYC-dependent transcription [33]. In contrast, other studies showed that SNF5 inhibits MYC function by tempering MYC’s ability to bind chromatin, resulting in decreased target gene transcription [48,49]. We uncovered an antagonistic role between MYC and SMARCB1 in controlling gene expression programs in insect vectors. Moreover, ChIP and EMSA analyses revealed that autophagy may be involved in SMARCB1’s effect on the antiviral response, identifying ATG5 as a transcriptional target of SMARCB1. RNAi experiments further confirmed that SMARCB1 inhibits the transcriptional levels of ATG5 and plays a major role in the antiviral pathway. In this study, we found that NcSMARCB1 has a dual function in decreasing the transcription level of ATGs by binding the promoter of ATG5 (GTGAGTCA) and inhibiting NcMYC’s ability to bind to the promoter of ATG5 (CACATG) and ATG8 (CACGTG), thereby disrupting the autophagy activation.

**Fig 7:**
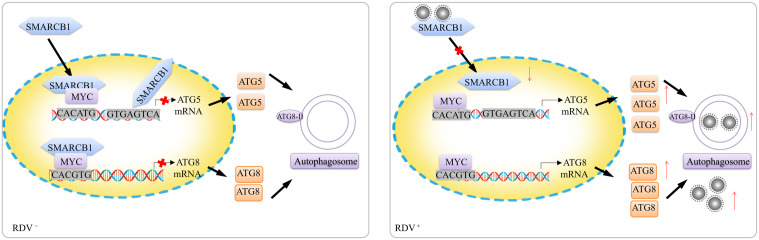
The model of RDV activating the autophagy pathway by obstructing SMARCB1 entry into the nucleus. MYC transcription factor regulates the expression of autophagy proteins ATG5 and ATG8 by directly targeting their promoters. NcSMARCB1 has a dual function in decreasing the transcription level of ATG5 by binding the ATG5 promoter and inhibiting the NcMYC’ ability to bind to promoter of ATG5 and ATG8, thus disrupting the autophagy activation. RDV major outer capsid protein P8 blocks the nuclear translocation of SMARCB1, disrupting the SMARCB1-MYC interaction and thereby relieving the transcriptional inhibition of ATG5 and ATG8, which leads to autophagy activation and for it’s persistent transmission.

According to Y2H and pull-down assays, we identified that NcSMARCB1 interacts with RDV P8. NcSMARCB1 effectively inhibit RDV P8 accumulation in *N. cincticeps*, while RDV decreases the expression of NcSMARCB1 to benefit its own accumulation. Previous study found that the Newcastle disease virus (NDV) yields were significantly higher after inhibition of BAF47, suggesting that chromatin-remodeling BAF complex mediates cellular antiviral activities in HeLa cells [47]. Our study for the first time showed that NcSMARCB1 plays an antiviral role in the insect vectors. Furthermore, we found that RDV has acquired the ability to inhibit leafhopper NcSMARCB1-NcMYC-ATG5 signaling by targeting NcSMARCB1. RDV binds to NcSMARCB1 and inhibits its nuclear translocation, resulting in upregulation of the NcSMARCB1-inhibited effector gene ATG5 and ATG8, thereby facilitating its persistent transmission (Fig 7). Similarly, the plant reovirus RGDV P8 also binds to RdSMARCB1 and inhibits its nuclear translocation, leading to the activation of autophagy pathway. In addition, RDV may also influence the transcription factor of SMARCB1 and reduce its transcriptional level, thereby inhibiting the antiviral effects of SMARCB1. The specific mechanisms involved warrant further investigation in the future. Previous study showed SMARCB1 also palys an important role in antiviral responses in Hela cells [47]. Thus, our results not only indicate that plant viruses have evolved tactics to manipulate the SMARCB1-MYC-autophagy signaling in their insect vectors but also suggest that mammalian viruses may utilize similar mechanisms to impair the host/vector’s SMARCB1 mediated antiviral responses.

## Methods

### Insects, viruses, and antibodies

Nonviruliferous *N. cincticeps* populations were collected from Fujian Province, China and propagated for several generations at 25 ± 3 °C in laboratory. RDV-infected rice plants were originally collected from Yunnan Province, China and maintained on rice plants via transmission by *N. cincticeps*. Non-viruliferous *R. dorsalis* individuals and RGDV-infected rice plants were collected from Guangdong Province, China. Rabbit polyclonal antibodies against RGDV P8, GAPDH, ATG8, ATG5, LAMP1, and SQSTM1 antigens were produced by GenScript Biotech Corporation, Nanjing, China [22,25,26]. Rabbit polyclonal antibodies against RDV P8, NcMYC were also prepared by Genscript Biotech Corporation, Nanjing, China. Rabbit or mouse polyclonal antibody against SMARCB1 were purchased from Proteintech (China). Specific antibodies against RDV P8 and SMARCB1 were conjugated to fluorescein isothiocyanate (FITC) or rhodamine to generate RDV P8-FITC, SMARCB1-rhodamine, respectively. Mouse monoclonal antibody against GST or His, and rabbit polyclonal antibody against histone H3, His, HA, Flag or Strep was purchased from Transgene Biotech (China). The actin dyes Alexa Fluor 647 Phalloidin and the nuclear dye 4’,6-diamidino-2-phenylindole (DAPI) were purchased from Thermo Fisher Scientific (Thermo Fisher Scientific, Roskilde, Denmark).

### Immunofluorescence assay

To clarify the distribution of NcSMARCB1 and RDV P8 in RDV-infected *N. cincticeps*, the intestines were dissected from 30 nonviruliferous and RDV-infection *N. cincticeps*. The samples were fixed in 4% (v/v) paraformaldehyde in PBS for 2 h, and then permeabilized in 2% (v/v) Triton-X for 1 h. The samples were then incubated with NcSMARCB1-rhodamine and RDV P8-FITC antibody (0.5 μg/μl). The DAPI was used to aid in identifying various tissues. Immunostained tissues were visualized using a laser scanning confocal microscope (LSCM, Leica TCS SPE).

### Immunoelectron microscopy

For immunoelectron microscopy, the excised intestines were fixed in 4% (v/v) paraformaldehyde in PBS for 3 h at 4 ºC, and after sequential dehydration in 30%, 50%, 70%, 90% and 100% alcohol, finally embedded in LR Gold Resin (Bioscience). The specimen was sectioned on an ultramicrotome (Leica), then incubated in blocking buffer (goat serum, 1:100) for 30 min. To observed the distribution of NcSMARCB1 and association of RDV and NcSMARCB1 in RDV-infected *N. cincticeps*, the sections were then incubated with antibodies in the following order: anti-SMARCB1 rabbit serum (1:200) for 2 h, 15-nm gold-conjugated goat-anti-rabbit IgG (1:50) for 2 h.

### Y2H assay

The cDNA library derived from *N. cincticeps* or the NcSMARCB1, NcMYC, RdSMARCB1 genes were constructed in the pGADT7 vector for prey plasmids. Full-length ORF of RDV P8, RGDV P8 was cloned in the pGBKT7 vector as a bait plasmid, which was then used to transform yeast strain AH109 to confirm the absence of self-activation. Thereafter, the cDNA library prey and RDV P8 bait were used to cotransform AH109, and transformants were dropped on the DDO (SD/-Trp-Leu), TDO (SD/-His/-Leu/-Trp) and QDO (SD/-Trp-Leu-His-Ade) culture medium and incubated at 30°C for 3–5 d. Positive clones were selected on QDO/X plates containing X-α-Gal (20 μg/mL) to detect galactosidase activity. The positive control pGBKT7–53/pGADT7-T and negative control pGBKT7-Lam/pGADT7-T were transformed in the same way. The primers used were shown in S1 Table.

### GST pull-down assay

The full-length ORFs of RDV P8, RGDV P8, NcSMARCB1, RdSMARCB1 and NcMYC were cloned into the vector pGEX-4T-3 or pET28a to express GST-RDV P8, GST-RGDV P8, GST-NcSMARCB1, His-NcSMARCB1, His-RdSMARCB1, His-NcMYC, respectively. Fusion proteins were expressed in the *Escherichia coli* strain BL21 by induction of 0.1 mM Isopropyl-D1-Thiogalactopyranoside at 16°C for 10 h. For GST pull-down assays to identify the interaction of RDV P8/NcSMARCB1 and NcSMARCB1/NcMYC, GST, GST-tag-fused proteins were incubated with 50 µL glutathione Sepharose4B beads (GE Healthcare, USA) at 4°C for 2 h. The bead bound proteins were washed five times and then incubated with His-tag-fused proteins for 4 h at 4°C. After being centrifuged and washed five times, the mixed proteins were detected by western blotting assay with His-tag and GST-tag antibodies. The primers used were shown in S1 Table.

### Co-IP assay

The total protein of 100 leafhopper *N. cincticeps* was extracted using cell lysis buffer (Promega). SMARCB1 mouse antibody was incubated with leafhopper protein for 4 h at 4°C. Then protein A/G-agarose beads were added and incubated for an additional 2 h at 4°C. In addition, proteins were incubated with IgG as negative control. After washing five times with lysis buffer, immunoprecipitated proteins were boiled in loading buffer for 5 min and detected by western blot with anti-SMARCB1 and NcMYC rabbit antibody. The experiments were performed three times.

### Expression analysis of NcSMARCB1 and NcMYC in *N. cincticeps* during RDV infection

To analyze the expression levels of NcSMARCB1 and NcMYC in healthy and RDV-infected *N. cincticeps*, insect total RNAs and proteins were extracted from 30 healthy and RDV-infected (4 days) *N. cincticeps*. The relative transcript and protein expression levels of NcSMARCB1 and NcMYC were detected by RT-qPCR and western blot assays. For RT-qPCR, total RNA form 30 leafhoppers was isolated using TRIzol reagent (Invitrogen) according to the manufacturer’s instructions. cDNA was synthesized using reverse transcriptase (Promega). qPCR was carried out using an ABI 7500 real-time PCR system (Applied Biosystems) with SYBR Green detection (Genestar). The cycling programme for all amplifications was 94°C for 2 min, followed by 40 cycles of 94°C for 10 s and 60°C for 30 s. The EF1 gene was quantified as an endogenous control and all primers were listed in S1 Table. All experiments were repeated three times independently. All qPCR data were calculated using the 2^−ΔΔCt^ method and differences were statistically evaluated using Student’s t test.

In the western blot assay, the concentration of total protein was determined with a BCA protein assay kit (Pierce, Rockford, USA) and equal amounts of protein from each sample were separated on a 12% SDS‒PAGE gel and subsequently transferred onto a PVDF membrane. The membranes were blocked with 5% skim milk in PBS containing Tween 20 (PBST) for 2 hours at room temperature. Following blocking, the membranes were incubated with the primary antibody diluted 1:2000 at 37°C for 2 hours. After three washes with PBST, the membranes were incubated with a horseradish peroxidase (HRP)-conjugated secondary antibody, also diluted 1:10000, at 37°C for 2 hours. Immunoreactive bands were visualized using a commercial enhanced chemiluminescence (ECL) detection system. Insect GAPDH was detected with GAPDH-specific IgG as a control. Quantification of western blot bands was performed using ImageJ software (version 1.8.0, Java 8). The band intensity ratios presented were derived from the blots shown in the corresponding figures. Uncropped original scans of all blots are provided as a supplementary figure in the Supplementary Information. All the experiments were performed three times.

To analyze the expression levels of RdSMARCB1 in healthy and RGDV-infected *N. cincticeps,* the process is same as above. Insect H3 was detected with tubulin-specific IgG as a control.

### Effect of *in vitro* synthesized dsRNAs on viral infection in leafhopper

RNA inference was carried out to suppress the expression of related genes and confirm the function on viral propagation and transmission by insect vectors. The T7 promoter with the sequence 5′-ATTCTCTAGAAGCTTAATACGACTCACTATAGGG-3′ was added to the forward and reverse primers to amplify a partial region of ~500–800 bp of NcSMARCB1, NcMYC, or GFP gene (S1 Table). The dsRNAs targeting NcSMARCB1 (dsNcSMARCB1), NcMYC (dsNcMYC,) or GFP (dsGFP) were synthesized according to the protocol for the T7 RiboMAX Express RNAi System kit (Promega, P1700).

To test the suppression of NcSMARCB1 or NcMYC expression on RDV infection in *N. cincticeps*, viruliferous second-instar *N. cincticeps* individuals were fed on RDV-infected rice plants for two days and then were microinjected with 23 nl dsNcSMARCB1, dsNcMYC RdE3 (3 μg/μl) or with dsGFP (3 μg/μl) as a control using an Auto-Nanoliter Injector (Drummond). The dsRNAs-treated *N. cincticeps* were transferred to healthy rice seedlings and approximately 100 treated leafhoppers were harvested at 4-day padp. The total RNAs were extracted from each of the leafhoppers, and RT-PCR assay was performed to confirm that they were viruliferous. Furthermore, total RNAs from 30 leafhoppers were extracted to estimate dsNcSMARCB1, dsNcMYC transcript levels and RDV titers by RT-qPCR assays. Total proteins were extracted from 30 leafhoppers to quantify the accumulation levels of NcSMARCB1, NcMYC and RDV P8 using a western blot assay.

To examine the function of NcSMARCB1 or NcMYC on the transmission efficiency of RDV by *N. cincticeps*, 50 dsRNAs-treated leafhopper individuals were placed in tubes that contained a single rice seedling for a 48‐h inoculation access period. After 21 days, the rice plants were surveyed for disease symptoms and subjected to RT-PCR testing for RDV.

### Baculovirus expression assay

To confirm the distribution of RDV P8, NcSMARCB1 and NcMYC in Sf9 cells, the baculovirus system was used to express RDV P8, NcSMARCB1 and NcMYC. The ORFs of RDV P8, NcSMARCB1 and NcMYC were cloned into vector pFAST‐T1 with His, HA and strep tag respectively using the designed primers in S1 Table. The plasmids were transformed into *E. coli* DH10Bac to generate a recombinant bacmid. Recombinant bacmids containing RDV P8, NcSMARCB1 and NcMYC were transfected into Sf9 cells in the presence of Cellfectin II (Thermo Fisher Scientific, 10362100) according to the manufacturer’s instructions. After incubation for 48 h, the Sf9 cells growing on the coverslips were fixed, permeabilized, immunolabelled with His-FITC, HA-rhodamine, then processed for immunofluorescence microscopy. To confirm the distribution of RGDV P8, RdSMARCB1 in Sf9 cells, the ORFs of RGDV P8, RdSMARCB1 were cloned into vector pFAST‐T1 with His and HA tag respectively and did the same process as above.

### Nuclear-cytoplasmic separation assay

To study whether RDV affect the distribution of NcSMARCB1, Nuclear and cytoplasmic proteins were extracted from 100 nonviruliferous and RDV-infected *N. cincticeps* and Sf9 cells expressed NcSMARCB1 or NcSMARCB1 and RDV P8 and using a Nuclear and Cytoplasmic Extraction kit (Beyotime, Jiangsu, China). The samples were grindded and incubated with cytoplasmic extraction A and B reagents (volume ratio of 20:1) supplemented with a protease inhibitor cocktail (Thermo Fisher Scientific, Waltham, MA, USA) for 30 min. The homogenate was then centrifuged and the supernatant was retained as the first cytoplasmic protein fraction. Subsequently, the precipitate was resuspended by another 200 μL of cytoplasmic extraction reagent A. After incubating for 15 min, the homogenate was added with 10 μL of cytoplasmic extraction reagent B and centrifuged. The resulting supernatant was retained as the second cytoplasmic protein fraction. Both extracts were then mixed and used as cytoplasmic proteins. Subsequently, the precipitate was added with 50 μL of nuclear extraction reagent and vortexed for 30 min. At last, the supernatant was retained as the nuclear protein extract fraction after centrifugation. The cytoplasmic and nuclear protein were detected by NcSMARCB1 and RDV P8 antibody. The H3 and GAPDH were used as reference proteins for nuclear and cytoplasmic proteins, respectively (Abcam, Cambridge, UK).

### ChIP-qPCR analysis

ChIP was performed using a Chromatin Immunoprecipitation (ChIP) Assay Kit (Beyotime biotechnology, China) in accordance with the manufacturer’s instructions. One hundred fifth-instar nymphs from nonviruliferous and RDV-infected leafhoppers were ground into a power in liquid nitrogen, and the extracted protein was cross-linked in cross-linking buffer with 1% (v/v) formaldehyde for 15 min before being incubated with 125 mmol/L glycine for 5 min. Then, 1 × buffer A containing 0.5 mmol/L DTT and 1 × protease inhibitor cocktail (PIC) were added. The DNA was digested by micrococcal nuclease for 20 min at 37 °C and then incubated with 5 mmol/L EDTA for 10 min. The pelleted nuclei were sonicated and centrifugated, the supernatant was diluted with 1 × ChIP buffer (containing 1 × PIC). Immunoprecipitation was performed using anti-NcSMARCB1, anti-NcMYC, rabbit IgG together with ChIP-Grade Protein G Magnetic Beads at 4 °C overnight. After sequentially being washed with low salt buffer three times, high salt buffer three times, and TE buffer two times, DNA was eluted in 1 × ChIP elution buffer for 30 min at 65 °C followed by addition of proteinase K and another 2 h incubation at 65°C. The ChIP-enriched DNA was purified using spin columns following the manufacturer′s instructions before being used for qPCR analysis on ABI 7500. Independent ChIP-qPCR experiments were performed three times with similar results. The primer of ATG5 and ATG8 promoter used for ChIP-qPCR are listed in S1 Table.

### Yeast one-hybrid assays

The promoter fragment (2,000 bp) of ATG8 was amplified by PCR, and inserted into the pAbAi vector, linearized by the restriction enzyme *BstB*I and then transformed into *Y1HGold* yeast strain as the bait. The cDNA library screening was carried out according to the protocol in the Matchmaker Gold Yeast One-Hybrid Library Screening System Kit (Clontech, Mountain View, CA, USA). Single colony was selected and amplified by PCR for sequencing to identify interacting TFs. The sequence of each candidate was analyzed by using BLAST searches against NR database.

Y1H assay was used to verify the binding of NcMYC to the ATG5 and ATG8 promoter. The NcMYC CDS was cloned into the pGADT7 vector as preys. The ATG5 and ATG8 promoter were inserted into the pAbAi vector as baits. The DNA fragments with mutations (from CAC[A/G]TG to CAAAAA) were generated by overlapping PCR. The preys and baits, along with the positive control (pGAD-p53 + p53-AbAi) and negative control (pGADT7-AD+baits), were combined and co-transformed into yeast *Y1HGold* strain. Y1H assay was performed follow the yeast maker yeast transformation system 2 user manual (Clontech).

### Electrophoretic mobility shift assay

The recombinant proteins NcSMARCB1 were expressed in *E. coli* Rosetta (DE3) and purified with Ni resin according to the manufacturer’s instructions. ATG5 promoter probes were 5′-end-labeled with Cy5 (FAM). The labeled probes were incubated with purified NcSMARCB1 in a 20 μl binding reaction system (50 mM TrisHCl, pH 7.5, 5 mM EDTA, 10 mM MgCl2, 0.5 M NaCl, 5 mM DTT, 0.05 mg/mL poly [dI-dC] and 40% glycerol) at room temperature for 30 min. The unlabeled DNA probe was used as a competitor. The samples were subjected on a 6% native polyacrylamide gel in 0.5 × Tris-borate-EDTA (TBE) buffer for 1 h and finally visualized. The detailed probe information of the ATG5 and ATG8 promoter is shown in S1 Table.

### Dual-luciferase reporter assay

The transcriptional activity of the upstream sequence of the ATG5 and ATG8 gene was detected with luciferase reporter assay in transiently transfected *Spodoptera frugiperda* (Sf9) cells. The about 2000-bp ATG5/ATG8 promoter sequences were amplified and cloned pGL3 vector. The Renilla luciferase control reporter plasmid pRL-CMV was used as the internal control. The full-length coding sequence of NcSMARCB1 and NcMYC was amplified and cloned into expression vector pIB/V5-His (Invitrogen, San Diego, CA, USA). The primers used for this experiment are shown in S1 Table in the Supporting Information. The plasmids were transfected into Sf9 cells transiently. After 60h, cells were collected and examined for Firefly and Renilla luciferase activities using the Dual-Luciferase Reporter Assay. The relative firefly luciferase activity normalized against the Renilla luciferase activity was shown as the mean±SD of three independent transfections. Each experiment did three times.

### Quantification and statistical analysis

All quantitative data presented in the text and figures were analyzed with GraphPad Prism 8.0 (GraphPad Software, San Diego, CA, USA). Two-tailed Student’s t-tests (for comparisons between two groups) were conducted to analyze the quantitative data from RT-qPCR assays, ChIP-qPCR and dual-luciferase reporter assay. Multiple comparisons of the means (for comparisons over two groups) were conducted to analyze the quantitative data from dual-luciferase reporter assay using a one-way analysis of variance followed by Tukey’s honestly significant difference (HSD) test analysis. Any p-values less than 0.05 were taken statistically significant.

For RT-qPCR, ChIP-qPCR and dual-luciferase reporter assay, we did three biological and technical replicates. For Y2H assay, GST pull-down assay, Co-IP assay, Y1H assay, EMSA, nuclear-cytoplasmic separation assay and western blot assay, we did three biological replicates.

For RT-qPCR and western blot assay, 30 insects collected for every biological replicate. For transmission efficiency assay, 50 plants were calculated for every biological replicate. For ChIP-qPCR, Co-IP assay and nuclear-cytoplasmic separation assay, 100 insects were collected for every biological replicate.

## Supporting information

S1 Fig RDV transmission efficiencies by dsATG5 and dsATG8 (A) or 3-MA and rapamycin (B) or dsNcMYC (C) or dsNcSMARCB1 (D)-treated leafhopper. Efficiencies of RDV transmission to rice seedlings by single RDV-positive leafhoppers after the microinjection of dsATG5, dsATG8, dsNcMYC, dsNcSMARCB1, dsGFP, 3-MA or rapamycin, as calculated by the percentage of RT-PCR-positive plants out of the total number of tested plants. Data are presented as means (± SD) of three replicates, and each replicate contains 50 insects (two-tailed t test). *, *p* < 0.05; **, *p* < 0.01.
(TIF)

S2 Fig Characterization of NcMYC, NcSMARCB1 and RdSMARCB1 sequences. (A) Characterization of NcMYC containing the bHLHzip_Myc domain. (B) Characterization of NcSMARCB1 containing the WH_NTD_SMARCB1 and Snf5 domain. (C) Characterization of RdSMARCB1 containing the WH_NTD_SMARCB1 and Snf5 domain.
(TIF)

S3 Fig Survival rates of 100 RDV-infected leafhopper treated with dsGFP or dsNcMYC. (TIF)

S4 Fig EMSA assay showing that NcSMARCB1 interferes with the binding of NcMYC and promoters of ATG5 probe1 (“CACATG”-containing sequences) (A) and ATG8 probe (“CACGTG”-containing sequences) (B). (TIF)

S5 Fig EMSA assay showing that NcSMARCB1 binds the ATG5 promoter probe 2 (“GTGAGTCA”-containing sequences) while not ATG5 promoter probe1 (“CACATG”-containing sequences). (TIF)

S6 Fig Analysis of putative interactors of RDV P8 in Y2H screening. (A) Interaction frequency of more than 30 putative interactors of *N. cincticeps* from the Y2H system. (B) GO categories of the putative interactors of *N. cincticeps* on molecular function.
(TIF)

S7 Fig RDV P8 did not change the distribution of NcMYC. Nuclear-cytoplasmic separation assay showing the nuclear (n) and cytoplasmic (c) distribution of NcMYC in Sf9 cells singly expressing NcMYC, co-expressing NcMYC with NcSMARCB1, together or or triply expressing NcMYC, NcSMARCB1 and RDV P8. H3 and GAPDH antibodies reacted with the proteins of the nucleus and cytoplasm, respectively.
(TIF)

S1 Table List of oligonucleotide primers used in this study. (DOCX)

S1 Data The raw data used in the figures. (XLSX)

S1 File Uncropped scans of blots. (DOCX)
