# Supplementary figures and images for: Reoviruses hijack the SMARCB1-MYC transcriptional regulation complex to activate autophagy for persistent viral infection in leafhopper vector
Source: PLoS Pathog. 2025 Oct 9;21(10):e1013569. doi: 10.1371/journal.ppat.1013569 (PMC12510602; doi:10.1371/journal.ppat.1013569)

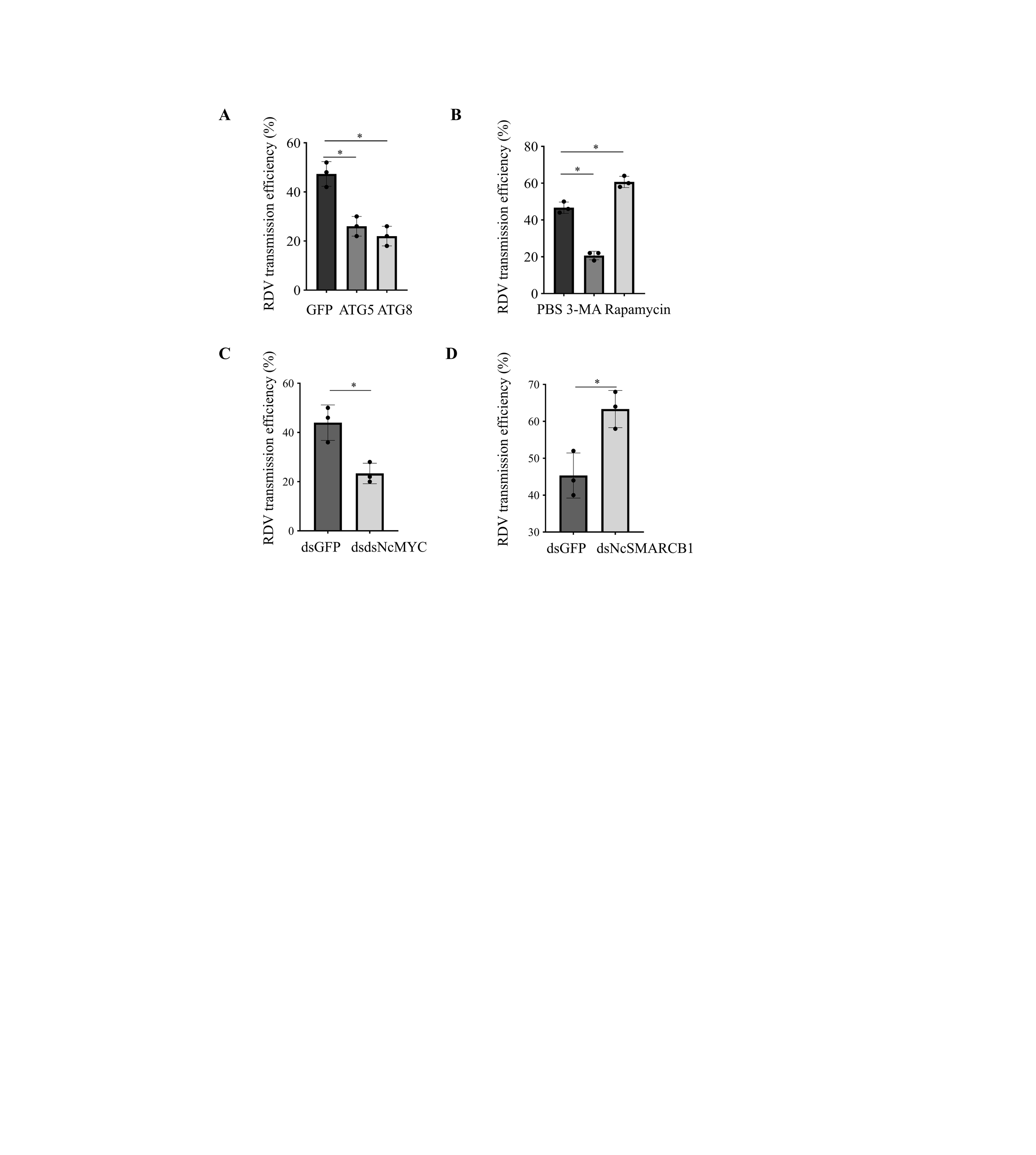

Supplement: S1 Fig — Efficiencies of RDV transmission to rice seedlings by single RDV-positive leafhoppers after the microinjection of dsATG5, dsATG8, dsNcMYC, dsNcSMARCB1, dsGFP, 3-MA or rapamycin, as calculated by the percentage of RT-PCR-positive plants out of the total number of tested plants. Data are presented as means (± SD) of three replicates, and each replicate contains 50 insects (two-tailed t test). *, p < 0.05; **, p < 0.01. (TIF) [file ppat.1013569.s001.tif]

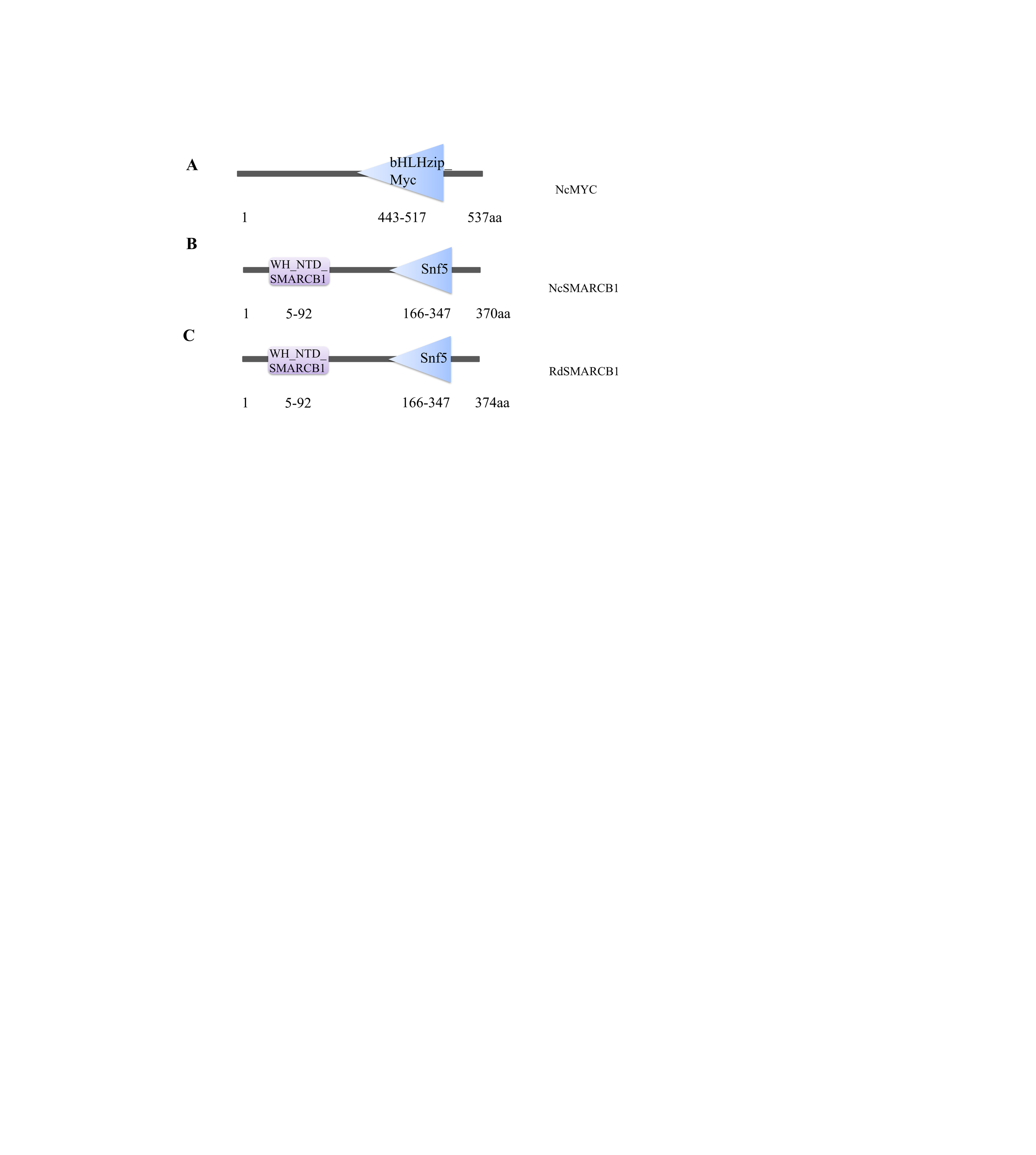

Supplement: S2 Fig — (A) Characterization of NcMYC containing the bHLHzip_Myc domain. (B) Characterization of NcSMARCB1 containing the WH_NTD_SMARCB1 and Snf5 domain. (C) Characterization of RdSMARCB1 containing the WH_NTD_SMARCB1 and Snf5 domain. (TIF) [file ppat.1013569.s002.tif]

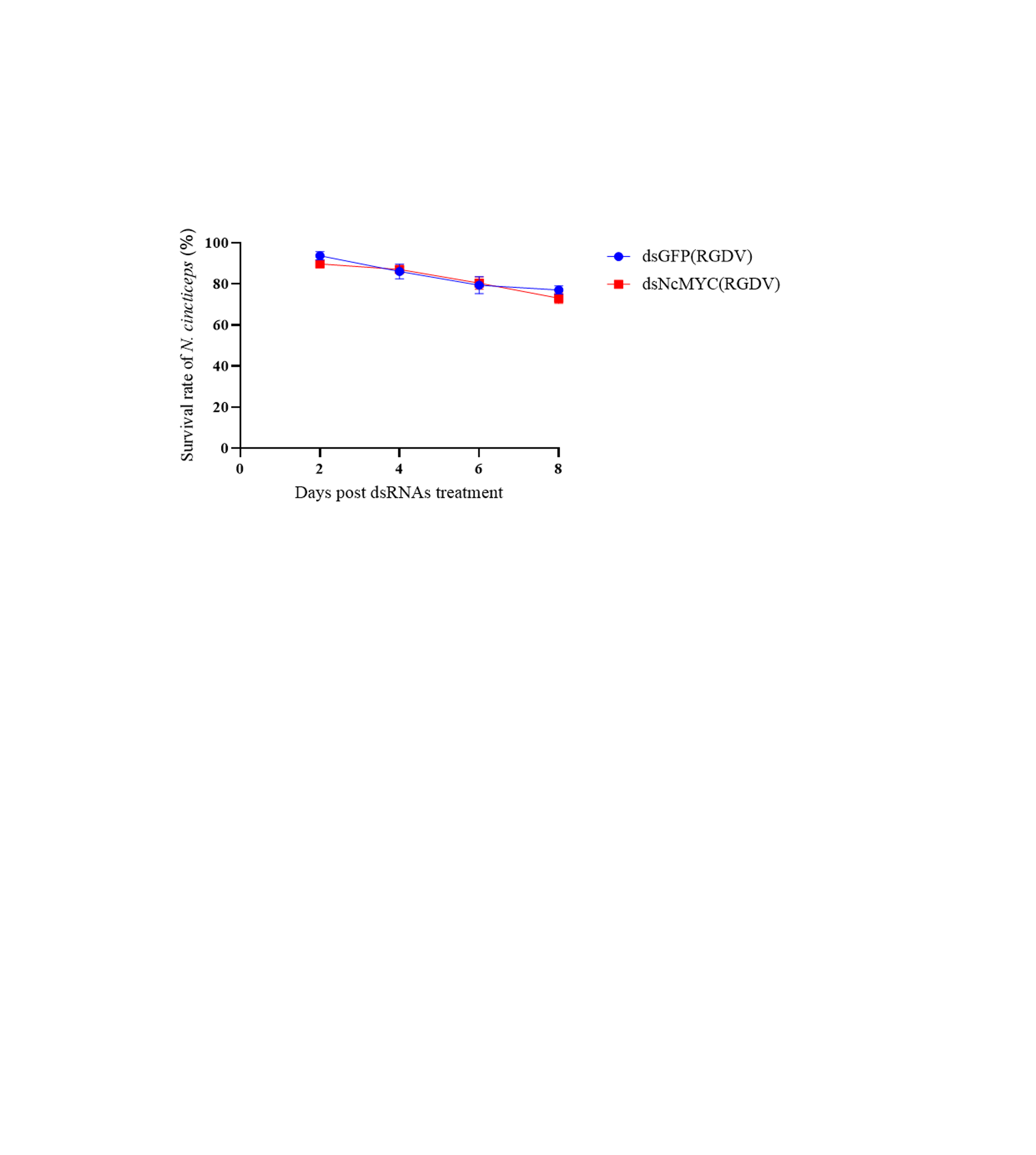

Supplement: S3 Fig — (TIF) [file ppat.1013569.s003.tif]

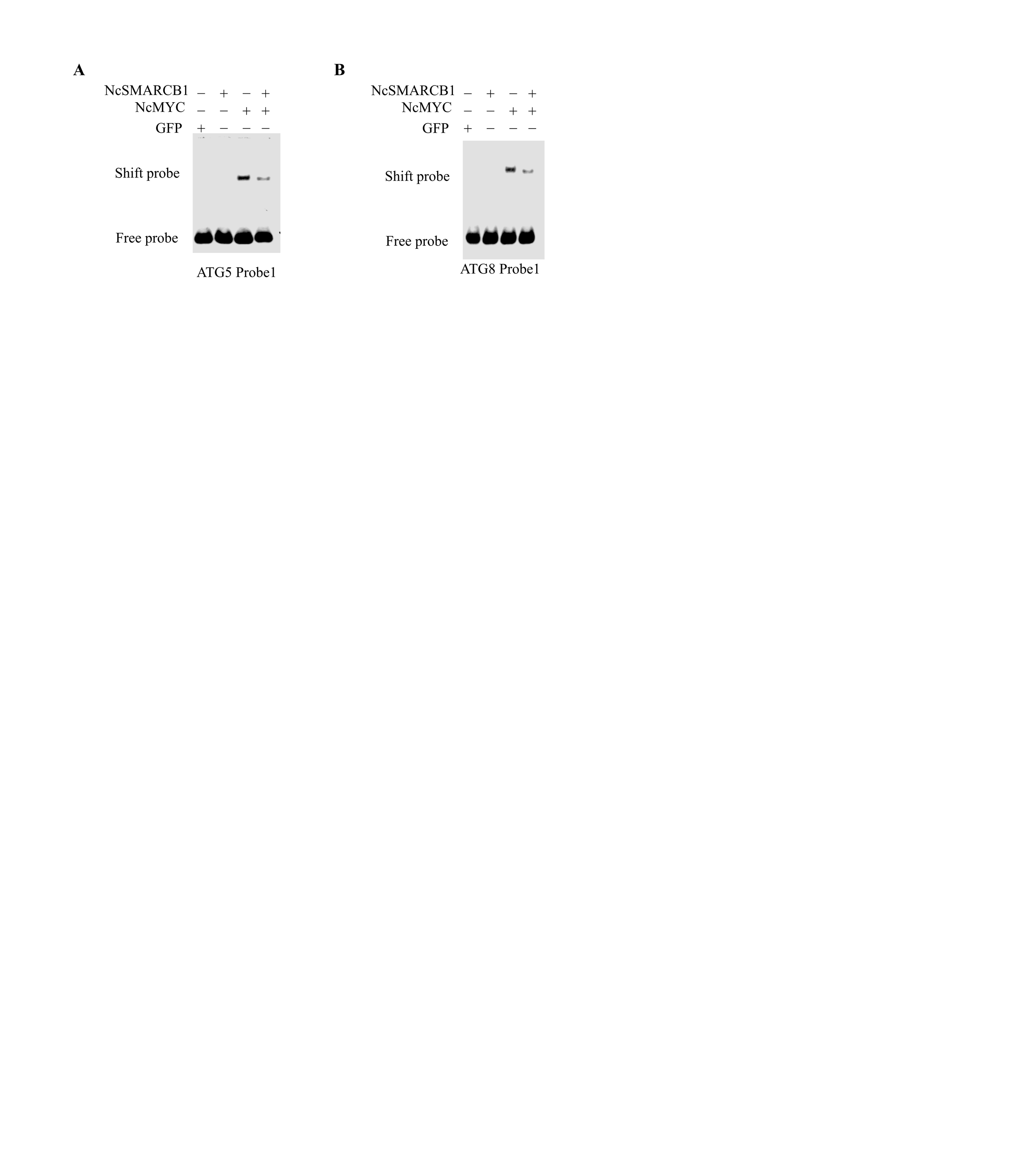

Supplement: S4 Fig — (TIF) [file ppat.1013569.s004.tif]

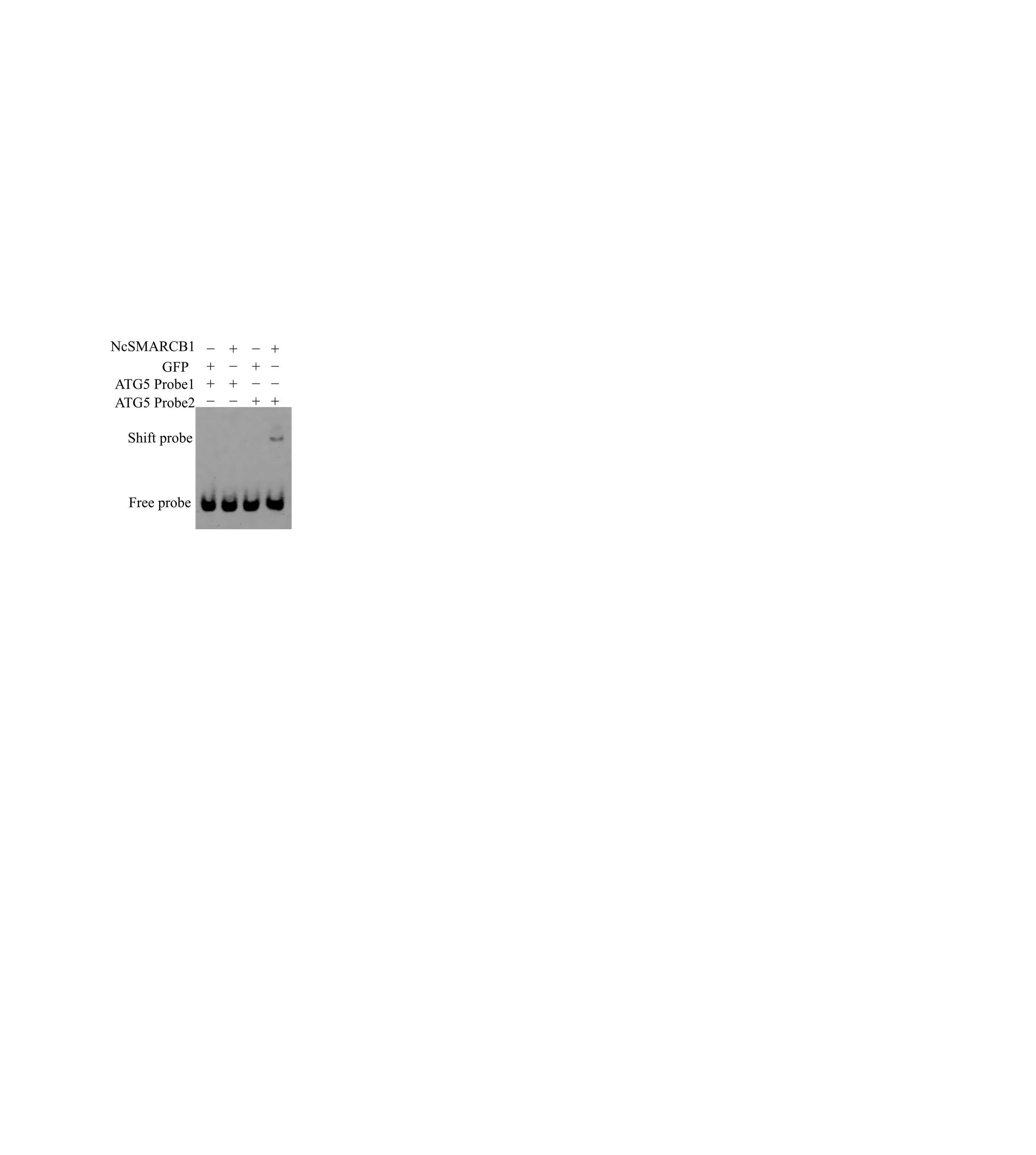

Supplement: S5 Fig — (TIF) [file ppat.1013569.s005.tif]

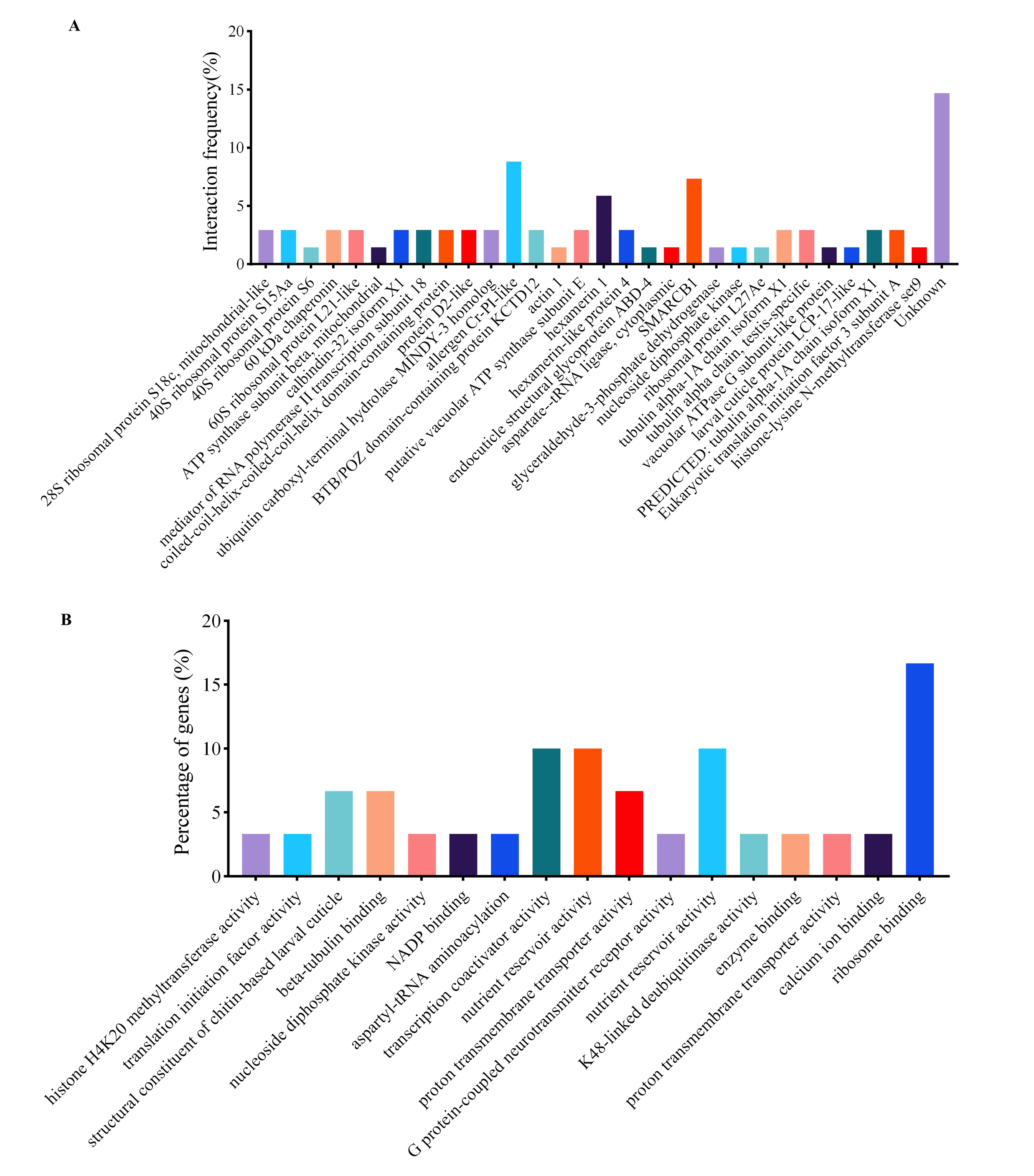

Supplement: S6 Fig — (A) Interaction frequency of more than 30 putative interactors of N. cincticeps from the Y2H system. (B) GO categories of the putative interactors of N. cincticeps on molecular function. (TIF) [file ppat.1013569.s006.tif]

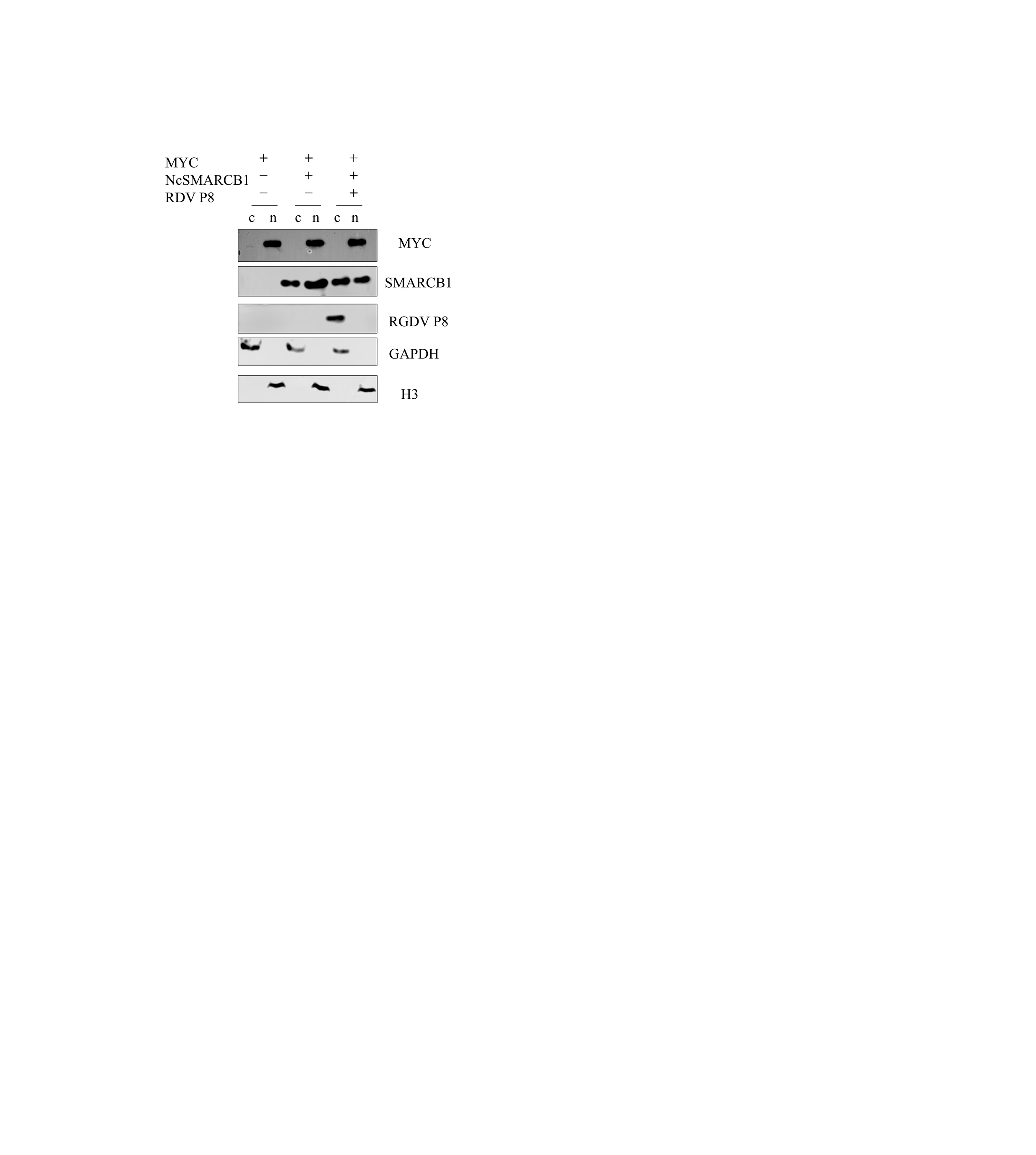

Supplement: S7 Fig — Nuclear-cytoplasmic separation assay showing the nuclear (n) and cytoplasmic (c) distribution of NcMYC in Sf9 cells singly expressing NcMYC, co-expressing NcMYC with NcSMARCB1, together or or triply expressing NcMYC, NcSMARCB1 and RDV P8. H3 and GAPDH antibodies reacted with the proteins of the nucleus and cytoplasm, respectively. (TIF) [file ppat.1013569.s007.tif]
