# Supplementary material for: Reoviruses hijack the SMARCB1-MYC transcriptional regulation complex to activate autophagy for persistent viral infection in leafhopper vector
Source: PLoS Pathog. 2025 Oct 9;21(10):e1013569. doi: 10.1371/journal.ppat.1013569 (PMC12510602; doi:10.1371/journal.ppat.1013569)
Supplement: S1 Table — (DOCX) [file ppat.1013569.s008.docx]

**Supplementary Table 1**. List of oligonucleotide primers used in this study.

| Oligonucleotide | Sequence (5'-3') |
| --- | --- |
| **Y2H** |  |
| BD-RDV-P8-F | tggccatggaggccgaattcATGTCACGCCAGATGTGGTTAGAC |
| BD-RDV-P8-R | gctgcaggtcgacggatcccATTTGGTCTATAGTATCTTCCAAATACGGCG |
| AD-NcSMARCB1-F | gccatggaggccagtgaattcATGGCCATAAGAACGTATGGAG |
| AD-NcSMARCB1-R | cagctcgagctcgatggatccTTATGTCCATCCTGGAGTGG |
| BD-NcSMARCB1-F | tggccatggaggccgaattcATGGCCATAAGAACGTATGGAG |
| BD-NcSMARCB1-R | gctgcaggtcgacggatcccTTATGTCCATCCTGGAGTGG |
| BD-NcMYC-F | tggccatggaggccgaattcATGCCGGCCTGCAAGATG |
| BD-NcMYC-R | gctgcaggtcgacggatcccGTTGAGATACTTGGCAGCGATG |
| AD-NcMYC-F | gccatggaggccagtgaattcATGCCGGCCTGCAAGATG |
| AD-NcMYC-R | cagctcgagctcgatggatccGTTGAGATACTTGGCAGCGATG |
| AD-RdSMARCB1F | ccatggaggccagtgaattcATGGCTATTAGAACGTATGGAG |
| AD-RdSMARCB1R | gctcgagctcgatggatcccTTAAACTATGTTTAAATTAGGCCTTAC |
| **expression vector in Sf9 cell** |  |
| pFast-RDV-P8-F | cccaccatcgggcgcggatccATGTCACGCCAGATGTGGTTAGAC |
| pFast-RDV-P8-His-R | tcgacgtaggcctttgaattcGTGATGATGATGATGATGATTTGGTCTATAGTATCTTCCAAATACGGCG |
| pFast-NcSMARCB1-F | cccaccatcgggcgcggatccATGGCCATAAGAACGTATGGAG |
| pFast-NcSMARCB1-HA-R | tcgacgtaggcctttgaattcttaagcgtaatctggaacatcgtatgggtATGTCCATCCTGGAGTGG |
| **expression vector in *E. coli*** |  |
| pGEX-RDV-P8-F | cgcgtggatccccgaattccATGTCACGCCAGATGTGGTTAGAC |
| pGEX-RDV-P8-R | tcacgatgcggccgctcgagATTTGGTCTATAGTATCTTCCAAATACGGCG |
| pGEX-NcMYC-F | cgcgtggatccccgaattccATGCCGGCCTGCAAGATG |
| pGEX-NcMYC-R | tcacgatgcggccgctcgagGTTGAGATACTTGGCAGCGATG |
| 28a-NcSMARCB1-F | atgggtcgcggatccgaattcATGGCCATAAGAACGTATGGAG |
| 28a-NcSMARCB1-R | gtggtggtggtggtgctcgagTTATGTCCATCCTGGAGTGG |
| 28a-RDV-P8-F | atgggtcgcggatccgaattcATGTCACGCCAGATGTGGTTAGAC |
| 28a-RDV-P8-R | tcacgatgcggccgctcgagATTTGGTCTATAGTATCTTCCAAATACGGCG |
| pGEX-RGDV P8-F | cgcgtggatccccgaattccATGTCGCGCCAAGCTTGG |
| pGEX-RGDV P8-R | tcacgatgcggccgctcgagTTAGTTTACTGTGTAATACCTAC |
| 28a-RdSMARCB1F | atgggtcgcggatccgaattcATGGCTATTAGAACGTATGGAG |
| 28a-RdSMARCB1R | tcacgatgcggccgctcgagTTAAACTATGTTTAAATTAGGCCTTAC |
|  |  |
| **Y1H** |  |
| pAbAi NcATG5-F | tcgagctcggtacccCTGTCTACTGTACAATGATGTAATTAGCTC |
| pAbAi NcATG5-R | ggtcgacagatccccCCGTACTTGTTAGTTGCAATTGC |
| pAbAi NcATG8-F | tcgagctcggtacccAAAGCATCTTCAGCCAAAATAGAC |
| pAbAi NcATG8-R | ggtcgacagatccccGGAGTGTTGAATAGATAAGTTTAAAG |
|  |  |
| **RNAi** |  |
| T7-NcSMARCB1-F | attctctagaagcttaatacgactcactatagggATGGCCATAAGAACGTATGGAG |
| T7-NcSMARCB1-R | attctctagaagcttaatacgactcactatagggGACGAATTCGCCTCCAAGACC |
| T7-NcATG5-F | attctctagaagcttaatacgactcactatagggGGACAACCTTTGAAATGGCATTACC |
| T7-NcATG5-R | attctctagaagcttaatacgactcactatagggTGAGTAGCCGACACAAAGATGCAAG |
| T7-NcATG8-F | attctctagaagcttaatacgactcactatagggATGAAATTTCAATACAAAGAAGAGCATCCATTTG |
| T7-NcATG8-R | attctctagaagcttaatacgactcactatagggAACTCGCTTGCGGATCAGGAAG |
|  |  |
| **RT-qPCR** |  |
| Q-RDV-P8-F | CAGACCCCGACATCGATTGAT |
| Q-RDV-P8-R | TGCCCTTTCAGAAAATCGAAACC |
| Q-NcEF1 F | CAGTGAGAGCCGTTTTGAG |
| Q-NcEF1 R | AGGGCATCTTGTCAGAGGGC |
| Q-NcSMARCB1-F | GCTCCCCTGCCAATCGTAG |
| Q-NcSMARCB1-R | TGTCCATCCTGGAGTGGTGT |
| Q-NcATG5-F | CGGAAAACACTACGCTGTCTC |
| Q-NcATG5-R | TGAGTAGCCGACACAAAGATGC |
| Q-NcATG8-F | CACCTCAGACCGGAAGATGC |
| Q-NcATG1-F | TGGGCCACCTGGAAGCTC |
| Q-NcATG1-R | CTTGTCAGCGGTAGTGCTGG |
| Q-NcATG4-F | CAAGCAATCTCTGGGACTGATTGG |
| Q-NcATG4-R | GAGCTTGCCCATCAGGTG |
| Q-NcATG6-F | CTTCTCACTGCCCTTGCAC |
| Q-NcATG6-R | CAGAGAATGAGTTTCCGGTGG |
| Q-NcATG12-F | GATATTCTTCTCAAACCTACAGCT |
| Q-NcATG12R | TGGCTAAGACAGTAGTGAAGAATC |
| Q-NcATG8-R | AGCATCACCATAGACGTTTTCATC |
| Q-RdSMARCB1-F | GAACAGTCCAGAGGCATTTG |
| Q-RdSMARCB1-R | GATGTCAAGGTCGCCCAG |
| Q-RdATG5-F | CAGGTTTCCGGAAGATGAACTC |
| Q-RdATG5-R | GATGGCCTTGCTCCGTCAG |
| Q-RdATG8-F | GTGATTGTGGAGAAGGCACC |
| Q-RdATG8-R | GACATTTTCATCACTGTATGCG |
| Q-RGDV-P8-F | GATTCAAGGGGCACAGAACG |
| Q-RGDV-P8-R | GTAATGGTTGCGACTGGGTC |
| Q-RdEF1 F | CCCTCTGACAAGATGCCCTG |
| Q-RdEF1 R | TTACGACCATACCGGGCTTG |
|  |  |
| **EMSA** |  |
| NcATG5-Prob1 | TTATAAGGAAAACTAGTTATTTTAGTTAAAAACGAAACACATGCATCACA |
| NcATG5-ProbM1 | TTATAAGGAAAACTAGTTATTTTAGTTAAAAACGAAACAAAAACATCACA |
| NcATG5-Prob2 | TTATGTTATGTATGTGAGTCAAGTGATCCGAAGCAATTGCAACTAACAAG |
| NcATG5-ProbM2 | TTATGTTATGTATGTTTTTAA AGTGATCCGAAGCAATTGCAACTAACAAG |
| NcATG8-Prob | CTCTCCTCCTTCCTCTCCGCTCCTCACCACGTGGGTTTGTTGAGTGGGAG |
| NcATG8-ProbM | CTCTCCTCCTTCCTCTCCGCTCCTCACCAAAAAGGTTTGTTGAGTGGGAG |
|  |  |
| **ChIP-qPCR** |  |
| Ch-IP-NcATG5F1 | GTCGATCCATAAGACCAAGAG |
| Ch-IP-NcATG5R1 | GGATTACCCCCAGGAGAG |
| Ch-IP-NcATG5F2 | GAGTCTGCCAATGCAAATAAG |
| Ch-IP-NcATG5R2 | CTTTGTGGATAACCCATCTC |
| Ch-IP-NcATG8F1 | ATGCACGGCGTCTTTCG |
| Ch-IP-NcATG8R1 | CTGCACCAATTAAAGGTTTTGTG |
|  |  |
| **Dual-luciferase reporter assay** |  |
| pIB-NcMYC-F | cgaatttaaagcttggtaccATGCCGGCCTGCAAGATG |
| pIB-NcMYC-R | cgaaccgcgggccctctagaGTTGAGATACTTGGCAGCGATG |
| pIB-NcSMARCB1-F | cgaatttaaagcttggtaccATGGCCATAAGAACGTATGGAG |
| pIB-NcSMARCB1-R | cgaaccgcgggccctctagaTTATGTCCATCCTGGAGTGG |
| pGL3-ATG5P-F | cgtgctagcccgggctcgagCTGTCTACTGTACAATGATGTAATTAGCTC |
| pGL3-ATG5P-R | agtaccggaatgccaagcttCCGTACTTGTTAGTTGCAATTGC |
| pGL3-ATG8P-F | cgtgctagcccgggctcgagAAAGCATCTTCAGCCAAAATAGAC |
| pGL3-ATG8P-R | agtaccggaatgccaagcttGGAGTGTTGAATAGATAAGTTTAAAG |
| BD-RGDV P8-F | tggccatggaggccgaattcATGTCGCGCCAAGCTTGG |
| BD-RGDV P8-R | gctgcaggtcgacggatcccTTAGTTTACTGTGTAATACCTAC |
|  |  |
