# Supplementary material for: Reoviruses hijack the SMARCB1-MYC transcriptional regulation complex to activate autophagy for persistent viral infection in leafhopper vector
Source: PLoS Pathog. 2025 Oct 9;21(10):e1013569. doi: 10.1371/journal.ppat.1013569 (PMC12510602; doi:10.1371/journal.ppat.1013569)

**Reoviruses hijack the SMARCB1-MYC transcriptional regulation complex to activate autophagy for persistent viral infection in leafhopper vector**

Hui Wang^a,^ ^☯^, Runfa Liu^a,^ ^☯^, Guangming Xiao^a^, Yanan Li^a^, Bozhong Li^a^，Qian Chen^a^，Taiyun Wei^a*^

^a^ State Key Laboratory of Agricultural and Forestry Biosecurity, Fujian Agriculture and Forestry University, Fuzhou, Fujian, China.

^☯^ These authors contributed equally: Hui Wang, Runfa Liu.

**Uncropped scans of blots**


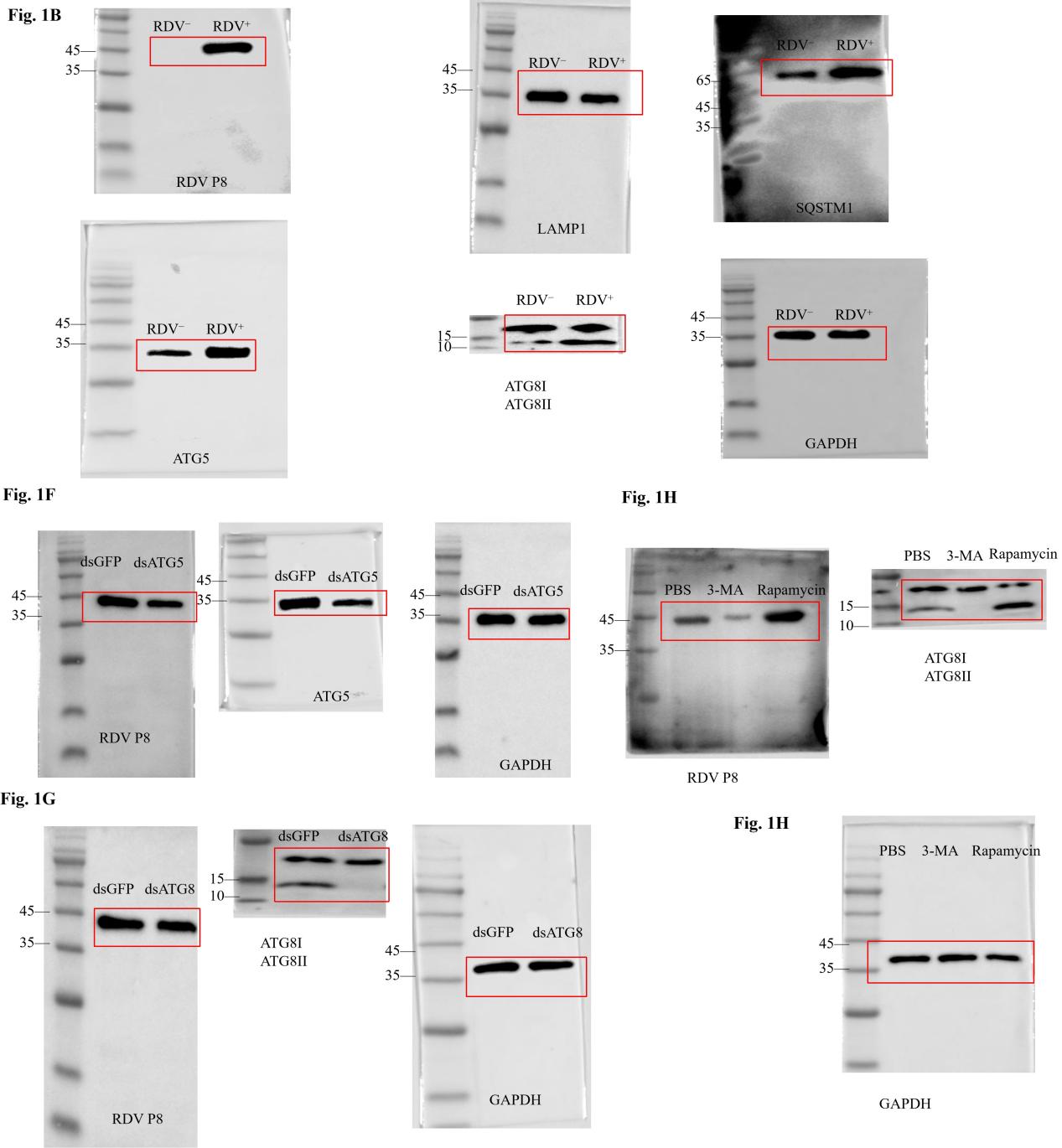


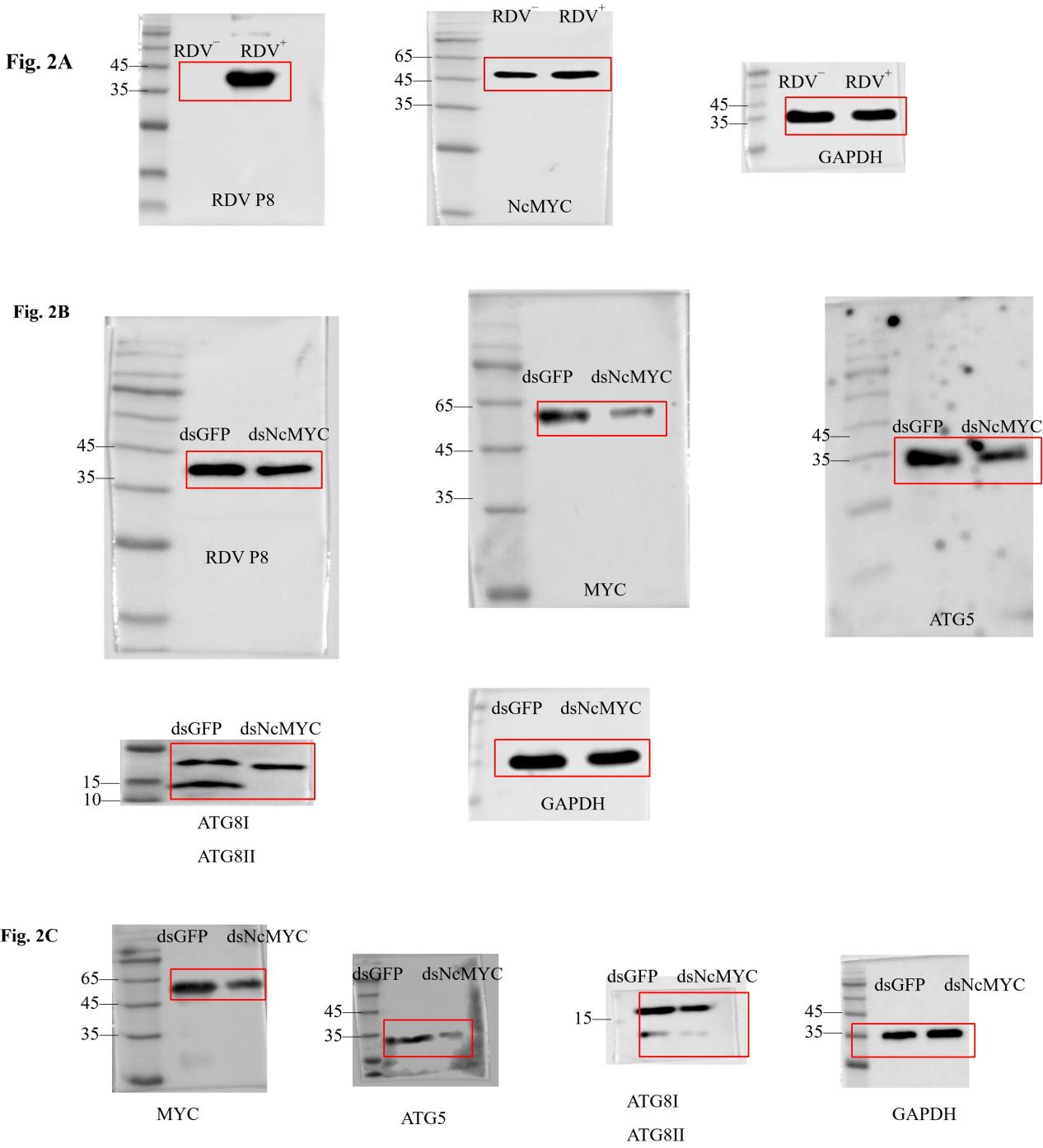

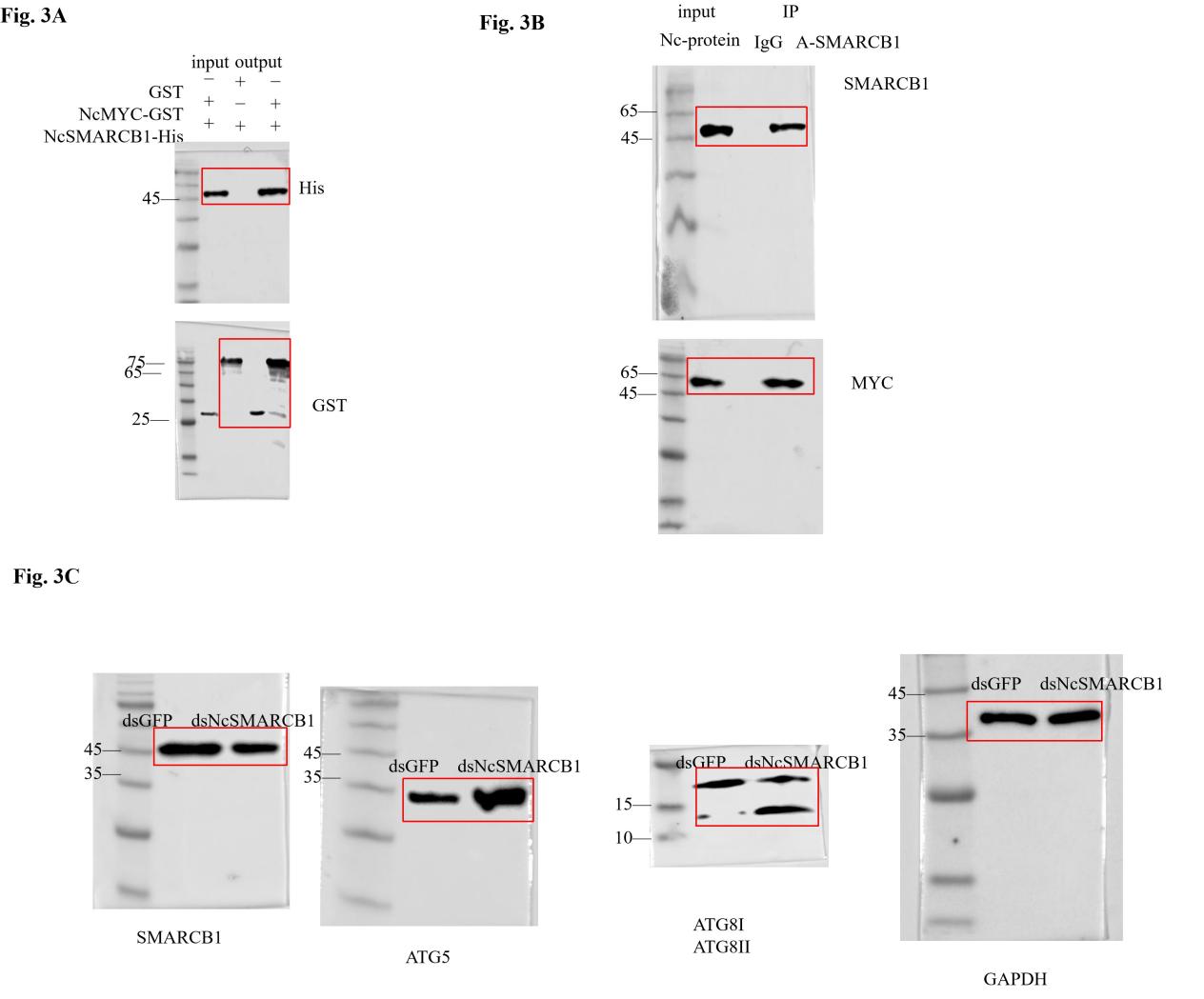

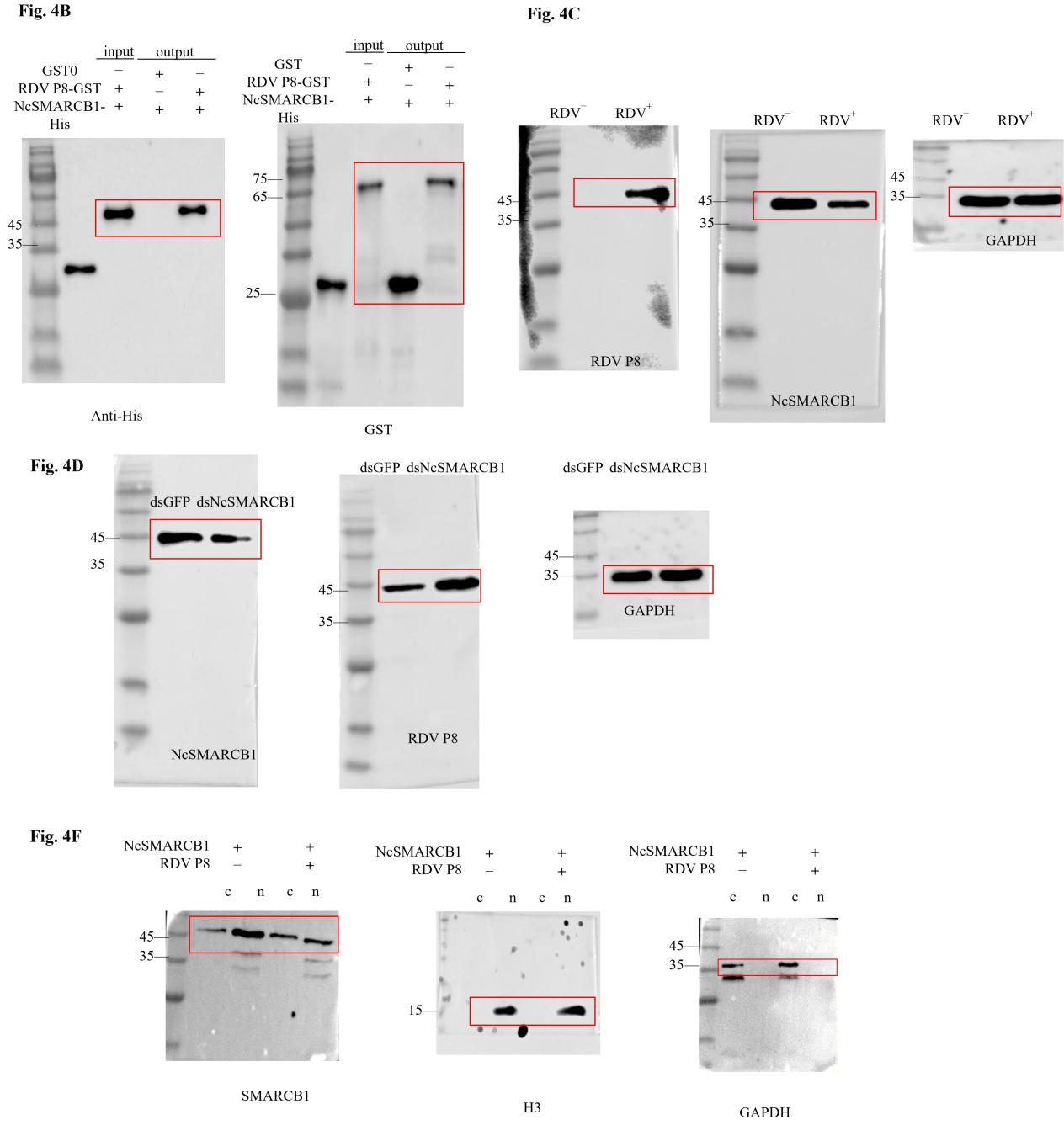


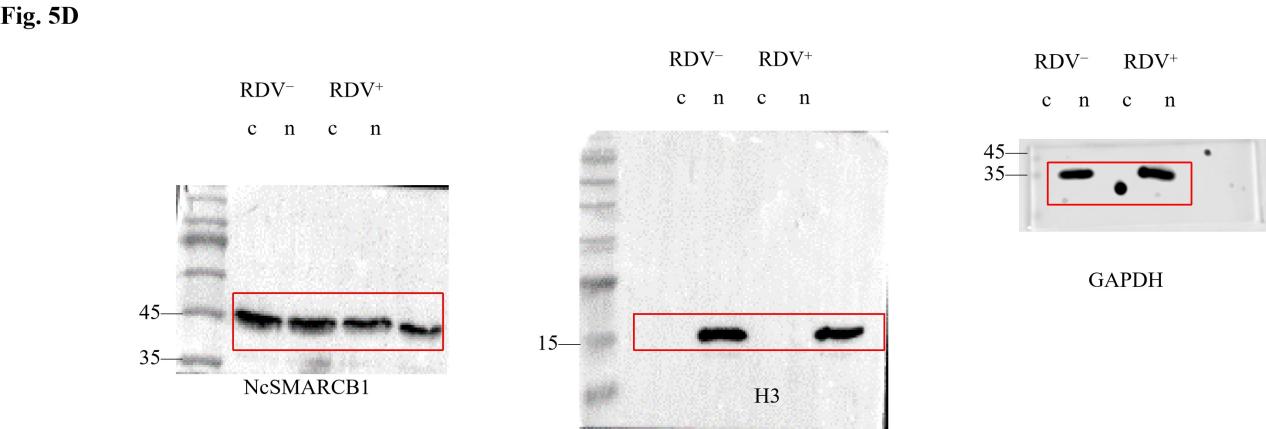

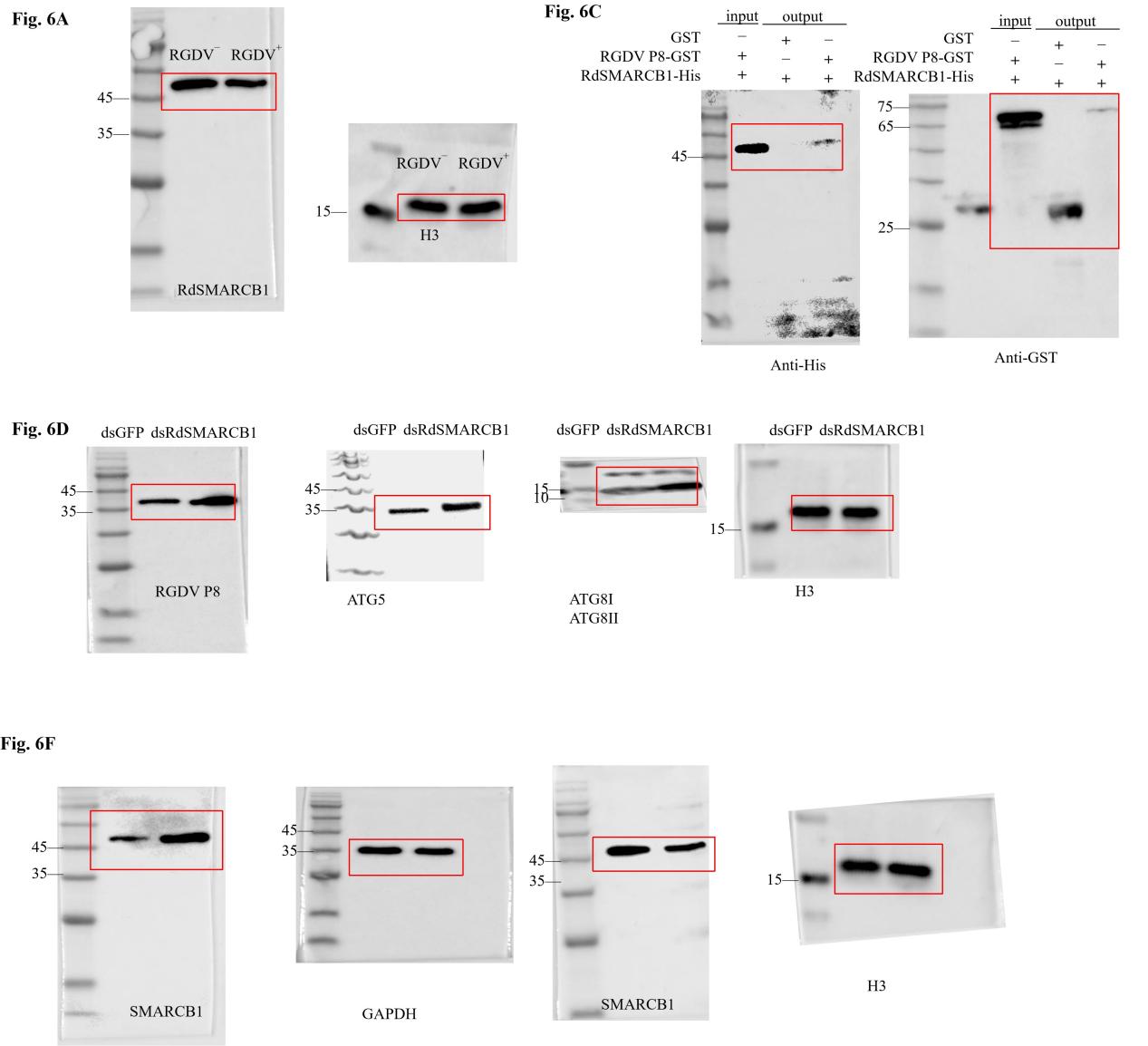

Supplement: S1 File — (DOCX) [file ppat.1013569.s010.docx]
